# Mapping an Extended Metabolic Profile of Gliomas Using High-Resolution ^31^P MRSI at 7T

**DOI:** 10.3389/fneur.2021.735071

**Published:** 2021-12-23

**Authors:** Andreas Korzowski, Nina Weckesser, Vanessa L. Franke, Johannes Breitling, Steffen Goerke, Heinz-Peter Schlemmer, Mark E. Ladd, Peter Bachert, Daniel Paech

**Affiliations:** ^1^Department of Medical Physics in Radiology, German Cancer Research Center (DKFZ), Heidelberg, Germany; ^2^Department of Radiology, German Cancer Research Center (DKFZ), Heidelberg, Germany; ^3^Faculty of Medicine, University of Heidelberg, Heidelberg, Germany; ^4^Faculty of Physics and Astronomy, University of Heidelberg, Heidelberg, Germany

**Keywords:** phosphorus, ^31^P, MRSI, 7T, UHF, brain, glioma, tumor

## Abstract

Phosphorus magnetic resonance spectroscopic imaging (^31^P MRSI) is of particular interest for investigations of patients with brain tumors as it enables to non-invasively assess altered energy and phospholipid metabolism *in vivo*. However, the limited sensitivity of ^31^P MRSI hampers its broader application at clinical field strengths. This study aimed to identify the additional value of ^31^P MRSI in patients with glioma at ultra-high *B*_0_ = 7T, where the increase in signal-to-noise ratio may foster its applicability for clinical research. High-quality, 3D ^31^P MRSI datasets with an effective voxel size of 5.7 ml were acquired from the brains of seven patients with newly diagnosed glioma. An optimized quantification model was implemented to reliably extract an extended metabolic profile, including low-concentrated metabolites such as extracellular inorganic phosphate, nicotinamide adenine dinucleotide [NAD(H)], and uridine diphosphoglucose (UDPG), which may act as novel tumor markers; a background signal was extracted as well, which affected measures of phosphomonoesters beneficially. Application of this model to the MRSI datasets yielded high-resolution maps of 12 different ^31^P metabolites, showing clear metabolic differences between white matter (WM) and gray matter, and between healthy and tumor tissues. Moreover, differences between tumor compartments in patients with high-grade glioma (HGG), i.e., gadolinium contrast-enhancing/necrotic regions (C+N) and peritumoral edema, could also be suggested from these maps. In the group of patients with HGG, the most significant changes in metabolite intensities were observed in C+N compared to WM, i.e., for phosphocholine +340%, UDPG +54%, glycerophosphoethanolamine −45%, and adenosine-5′-triphosphate −29%. Furthermore, a prominent signal from mobile phospholipids appeared in C+N. In the group of patients with low-grade glioma, only the NAD(H) intensity changed significantly by −28% in the tumor compared to WM. Besides the potential of ^31^P MRSI at 7T to provide novel insights into the biochemistry of gliomas *in vivo*, the attainable spatial resolutions improve the interpretability of ^31^P metabolite intensities obtained from malignant tissues, particularly when only subtle differences compared to healthy tissues are expected. In conclusion, this pilot study demonstrates that ^31^P MRSI at 7T has potential value for the clinical research of glioma.

## Introduction

Gliomas are the most common primary brain tumors in adults, with a generally poor prognosis due to persisting challenges in treatment (1). To improve our understanding of the underlying metabolic mechanisms in their pathogenesis, as well as to improve diagnosis and patient-specific decision making on therapeutic options, non-invasive imaging of metabolic abnormalities present in brain tumors, such as deregulated cellular energetics (2), increased membrane turnover (3), and associated alterations in tissue pH (4) play a crucial role. A well-suited option for non-invasive imaging of these characteristics is *in vivo* phosphorus magnetic resonance spectroscopic imaging (^31^P MRSI), as it provides a window into energy and phospholipid metabolism as well as tissue pH. As such, ^31^P MRS(I) has been applied to patients with glioma for some time in several studies at clinical field strengths *B*_0_ ≤ 3 T and has recently been shown to potentially predict therapy response *via* phospholipid metabolites (5) and the site of tumor progression *via* intracellular pH (6). Another recent study suggests that ^31^P MRSI could in principle also detect the mutation status of the isocitrate dehydrogenase (IDH) *via* phospholipid metabolites (7), further highlighting the potential of applying ^31^P MRSI in patients with glioma.

However, the limited sensitivity of *in vivo* ^31^P MRSI still hampers its broader application in clinical studies, particularly in the human brain where concentrations of ^31^P metabolites are relatively low. One of the challenges is the large effective voxel sizes applied at clinical field strength, typically on the order of about 50 ml at *B*_0_ = 3 T (8). These voxel sizes compromise the clear separation of healthy and tumor tissue spectra (5, 6, 9), even more so for the separation of metabolically different tumor compartments, which in turn complicates the interpretation of ^31^P MRSI results obtained in patients. The increase in signal-to-noise ratio (SNR) at ultra-high fields (UHF) *B*_0_ ≥ 7T may foster the clinical applicability of ^31^P MRSI by allowing the spatial resolution to be increased within clinically-relevant measurement times and/or by enabling the detection of even lower-concentrated metabolites. Additionally, the improved spectral resolution at higher fields leads to an increased specificity in metabolite assignment. In this regard, as an example, the improved tissue pH determination *via* the separate detection of intra- (P_i_) and extracellular inorganic phosphate (eP_i_) pools at UHF (10) has already demonstrated a potential added value for the application to glioma. Up to now, however, only singular ^31^P MRSI datasets of brain tumors at UHF have been presented, e.g., in (11), and the detection of other potentially interesting signals like low-concentrated Nicotinamide Adenine Dinucleotide [NAD(H)] and Uridine Diphosphoglucose (UDPG), which has recently been described in the healthy human brain (12–15), has not yet been tested in tumor tissue. Thus, ^31^P MRSI at UHF may also provide novel insights into tumor metabolism.

The aim of this study is to identify the additional value of ^31^P MRSI at UHF for application in clinical research of brain tumors in terms of (I) the detection of novel tumor markers and (II) the improved interpretability of results *via* increased spatial resolution. For this purpose, we acquired 3D ^31^P MRSI datasets in a small cohort of patients with newly diagnosed glioma at *B*_0_ = 7T with effective voxel sizes of 5.7 ml that enabled a good separation of individual tissues. A robust, optimized quantification model was implemented to extract an extended ^31^P metabolic profile from these datasets, including low-concentrated metabolites such as eP_i_, NAD(H), and UDPG, a signal attributed to mobile phospholipids (MPL), as well as a background signal underlying the phosphomonoester (PME) resonances. This model enabled high-resolution 3D maps of 12 different ^31^P metabolites to be generated, which were analyzed for regional differences in the individuals, not only between healthy and diseased tissues, but also between diseased tissue compartments. Finally, a group analysis revealed the most interesting changes that indicate malignant tissues.

## Materials and Methods

### Experimental Setup and Data Processing

Seven patients with newly diagnosed adult-type diffuse gliomas [according to the 2021 WHO classification (16): 2 × astrocytoma, IDH-mutant, CNS WHO grade 2, 1 × astrocytoma, IDH-mutant, CNS WHO grade 3, 4 × glioblastoma, IDH-wildtype, CNS WHO grade 4; 4 males; age: 23–78] were included in this study. In addition, measurements from three healthy volunteers (2 males; age: 25–35) were included in this study for comparison reasons. All examinations were approved by the local ethics committee of the Medical Faculty of the University of Heidelberg, and written informed consent was received from all subjects.

**Figure 1:**
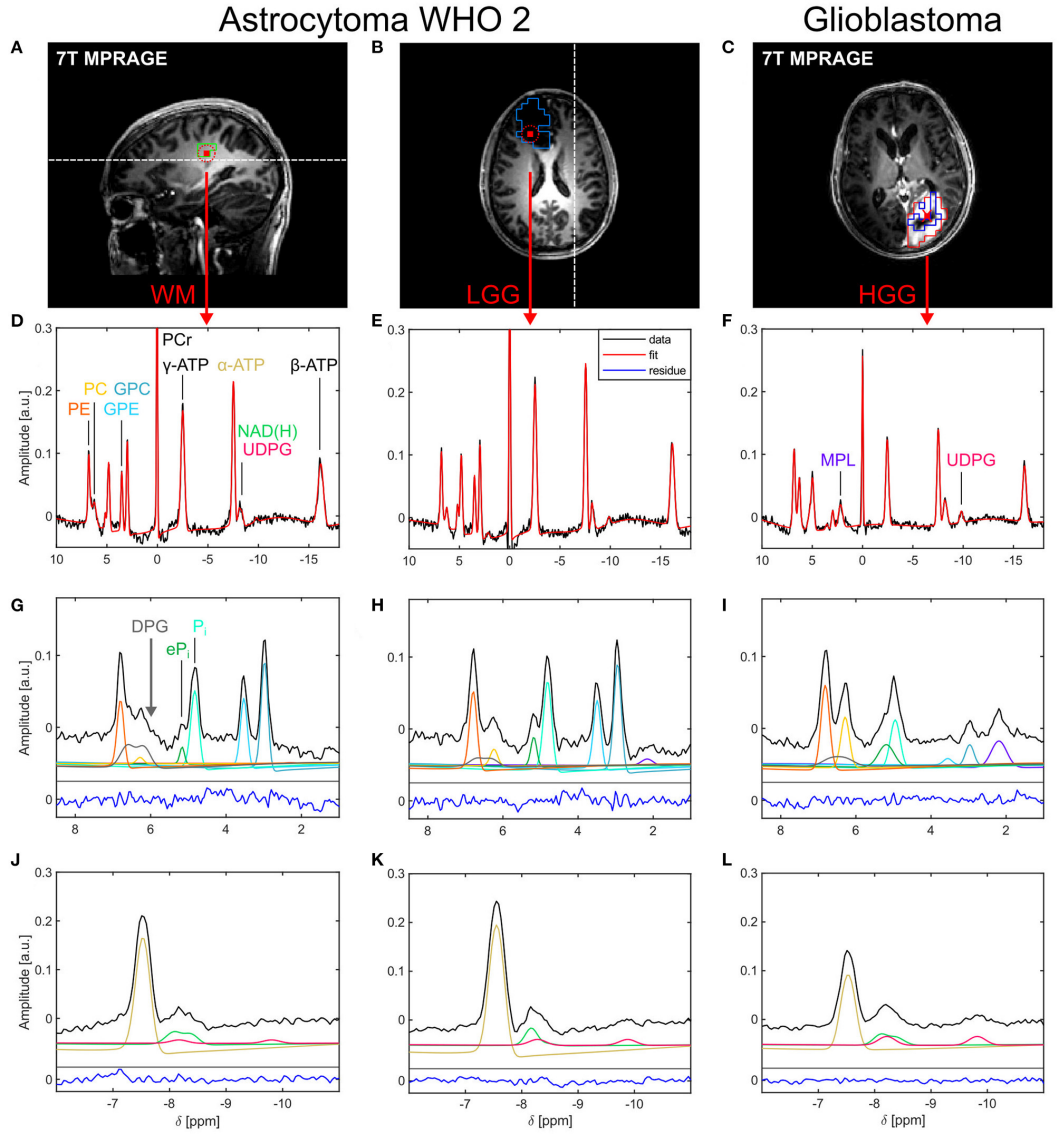
Representative localized ^31^P brain spectra and corresponding fits from a patient with a low-grade glioma [astrocytoma WHO 2; healthy tissue: **(A,D,G,J)**; tumor tissue: **(B,E,H,K)**] and a patient with a high-grade glioma [glioblastoma; tumor tissue only; **(C,F,I,L)**]. **(A–C)** Morphological images illustrating voxel positions [red squares in **(A,B)** indicate the interpolated nominal voxel volume and the red dashed circles the effective spherical voxel volume; red cross in **(C)**] and representative ROI definitions [green box in **(A)**: WM; light blue box in **(B)**: WTV; red box in **(C)**: GDCE; dark blue box in **(C)**: NEC]. The white dashed lines in **(A,B)** indicate the slice positions of **(B,A)**, respectively. **(D–F)** Acquired spectra (black lines) and corresponding fits (red lines) displayed over the full spectral range. Spectral excerpts from 1.0 to 8.5 ppm **(G–I)** and from −11.0 to −6.0 ppm **(J–L)** illustrate the individual fitted metabolite signals in these spectral regions (colored lines as indicated by the peak assignments in **(D,F,G)**; shifted on the y-axis for better visualization). The fitting residuals (blue lines) in **(G–L)** are scaled equally to the data. For visualization purposes, all spectra and fits were corrected for first-order phase with *t*_0_ = 1.1 ms.

The examinations utilized the high-resolution ^31^P brain MRSI protocol as described in detail in (10). In short, measurements were performed on a 7T whole-body MR scanner (Magnetom 7T; Siemens Healthineers, Erlangen, Germany) using a double-resonant ^31^P/^1^H phased-array head coil with 32 ^31^P receiver elements (Rapid Biomedical, Rimpar, Germany). A 3D CSI sequence (matrix size = (20 × 24 × 16), nominal isotropic resolution = (1.25 cm)3, *T*_R_ = 250 ms, α = 20°, Δ*f* = 5,000 Hz, 1,024 time points, Hamming-weighted k-space sampling with 18 averages of k-space center, transient ^31^P-{^1^H} nuclear Overhauser effect (NOE) enhancement (17) *via* adiabatic water proton inversion (hyperbolic secant shaped, *B*_1, mean_ ≈ 6 μT, *T*_Pulse_ = 6 ms, *T*_I_ = 10 ms prior to ^31^P excitation), acquisition duration = 51 min) was used to obtain high-SNR ^31^P MRSI datasets with small effective voxel size of 5.7 ml (spherical-shaped voxels; indicated by the red dashed circles in Figures 1A,B). 3D T1-weighted ^1^H images were acquired using an MPRAGE sequence [*T*_R_ = 3.4 s, *T*_I_ = 1.2 s, *T*_E_ = 1.46 ms, α = 6°, nominal resolution = (1.4 mm)3] for morphological imaging. All subjects have tolerated well the total examination duration of ~65 min.

Prior to the 7T examination, all patients underwent clinical routine imaging on a 3T MR scanner (Magnetom Verio; Siemens Healthineers, Erlangen, Germany). Clinical imaging included acquisition of multislice T2-weighted FLAIR [*T*_R_ = 8.5 s, *T*_I_ = 2.4 s, *T*_E_ = 135 ms, nominal resolution = (0.9 mm)^2^, slice thickness = 5 mm] and gadolinium contrast-enhanced 3D T1-weighted MPRAGE [*T*_R_ = 1.71 s, *T*_I_ = 1.1 s, *T*_E_ = 4.04 ms, α = 15°, nominal resolution = (0.5 mm)^2^ × 1.3 mm] images, both required for tumor segmentation.

The ^31^P MRSI datasets were processed and evaluated in MATLAB R2020b (The Mathworks, Natick, Massachusetts, USA) using customized scripts. First, the multichannel ^31^P MRSI data were reconstructed from the raw data files, and coil-combined using the *whitened SVD* algorithm (18). Afterwards, these datasets were denoised following the *Moderate Low-Rank Denoising* approach which is described in detail in (10). As in the earlier study, the rank of each low-rank approximated MRSI dataset was chosen to yield a specific SNR gain of 4, which enabled a reliable quantification of the low-concentrated metabolites as e.g. eP_i_ in most parts of the brain. Across all subjects, thresholding ranks for denoising were consistently determined to be between 30 and 32. Finally, the denoised datasets were further processed applying a 1-fold spatial zero-filling (interpolated matrix size of 40 × 48 × 32), and a 15-Hz Gaussian filter in the time-domain (chosen as a compromise between the observed ^31^P linewidths for the different metabolites), yielding the final ^31^P MRSI datasets.

### Quantification of ^31^P Spectra

^31^P spectra were quantified in the time-domain using an improved version of the home-built MATLAB implementation of the AMARES algorithm (19) described in (10). The time-domain data was fitted to the function describing *K* resonances modeled as Gaussian lines, with amplitude *a*_k_, damping *d*_k_, and frequency *f*_k_ for the *k*-th resonance, and the zero-order phase φ_0_. To model the resonances as multiplets where appropriate, the inclusion of linear relations between resonances was enabled in the algorithm. A begin time of *t*_0_ = 1.1 ms was applied in the modeling according to the MRSI acquisition.


(1) $$\begin{array}{l} \text{s}\left(\text{t}\right)=\sum_{\text{k}=1}^{\text{K}}{\text{a}}_{\text{k}}{\text{e}}^{\text{i}\varphi_{0}}{\text{e}}^{-{\text{d}}_{\text{k}}^{2}{\text{t}}^{2}}{\text{e}}^{2\text{πi}{\text{f}}_{\text{k}}\text{t}} \end{array}$$

As in the earlier study (10), quantification was performed in two steps: (I) prefitting and (II) main quantification of all detectable metabolites. The prefitting step for the estimation of the local *B*_0_ shift and φ_0_ by fitting the Phosphocreatine (PCr) resonance (as singlet, variable *f* = [−0.5, +0.5] ppm), however, was extended by simultaneously modeling the Glycerophosphocholine (GPC) resonance (as singlet, fixed Δ*f* = +2.95 ppm relative to PCr) and the α-Adenosine-5′-Triphosphate (ATP) resonance (as singlet, fixed Δ*f* = −7.56 ppm relative to PCr), which resulted in an improved robustness of the parameter estimation in voxels with significant signal contributions from muscle tissue. The main quantification step was then performed on the zero-order phase-corrected MRSI dataset, with frequency constraints adapted to the local *B*_0_ estimated in the prefitting step. Potential residual phase errors were compensated by allowing to fit φ_0_ = [−0.09 × π, +0.09 × π] in addition to the prefitting result.

Based on the signals identified in voxels with brain parenchyma of the acquired ^31^P MRSI datasets (cf. Figures 1D,F,G), the optimized quantification model described in Table 1 was implemented. This enabled to extract an extended profile of 12 ^31^P metabolites in the main quantification step, including PCr, ATP, NAD(H), GPC, Glycerophosphoethanolamine (GPE), Phosphocholine (PC), Phosphoethanolamine (PE), P_i_ and eP_i_, as well as UDPG, MPL, and 2,3-Diphosphoglycerate (DPG). UDPG was modeled as pseudo-doublet, based on the findings in (13, 14), with a fixed frequency spacing of Δ*f* = 1.6 ppm. To account for a potential variation in the metabolic composition of its resonances (14, 15), i.e., the different derivatives of UDPG, the upfield resonance was allowed to vary in the frequency range *f* = [−9.95, −9.60] ppm. Based on earlier reports attributing the signal in brain tissue around 2.2 ppm to mobile phospholipids (6, 8), the MPL resonance was modeled as a singlet with boundaries *f* = [+2.15, +2.25] ppm. To model background signals potentially underlying the PME resonances (20, 21), assumed here as a contribution of deoxygenated blood, DPG was included to the model as pseudo-doublet. To account for a potential variation in the blood oxygenation level, the upfield resonance was allowed to vary in the frequency range *f* = [+6.00, +6.30] ppm, with a fixed frequency spacing of Δ*f* = 0.4 ppm [estimated from the results of (22)]. Note that in contrast to the earlier model in (10) where no linear relations were applied, here, GPC, GPE, PC, and PE were constrained to have equal dampings. Moreover, NAD(H) was modeled as multiplet, with dampings coupled to the damping of α-ATP: the resonance of the oxidized form, NAD^+^, was modeled as quartet with spectral parameters derived according to (23) for *B*_0_ = 7T, and boundaries for the center frequency *f* = [−8.35, −8.27] ppm; the resonance of the reduced form, NADH, was modeled as singlet, with its intensity being allowed to vary within a range covering a redox ratio (RX = NAD^+^/NADH) of [0.3, 12]. Further note that within each (pseudo-)multiplet, equal dampings were constrained to each resonance. Finally, the inorganic phosphate resonances were modeled in this study utilizing linear relations based on the findings in (10): the P_i_ resonance was subject to the boundaries *f* = [+4.7, +5.0] ppm, while the eP_i_ resonance was subject to a variable frequency shift in the range of Δ*f* = [+0.1, +0.5] ppm relative to P_i_, corresponding roughly to the earlier defined excluded frequency range.

**Table 1:** Optimized parameters for the extended quantification model of ^31^P brain spectra.

**Metabolite**	**Multiplicity**	**Intensity ratios**	**Frequency boundaries [ppm]**	**Frequency shift constraints [ppm]**	**Damping boundaries** **× π [rad/s]**	**Damping constraints**
PCr	Singlet		[−0.05, +0.05]		[3, 50]	
γ-ATP	Doublet	1:1	[−2.60, −2.40] (center)	−0.07, +0.07 ^*^ (w.r.t. center)	[10, 70]	
α-ATP	Doublet	1:1	[−7.60, −7.50] (center)	−0.07, +0.07 ^*^ (w.r.t. center)	[10, 60]	
β-ATP	Triplet	1:2:1	[−16.30, −16.00] (center)	−0.14, 0.00, +0.14 ^*^ (w.r.t. center)	[15, 80]	
NAD^+^	Quartet	0.37:1:1:0.37	[−8.35, −8.27] (center)	−0.26, −0.10, +0.10, +0.26 (w.r.t. center)		Equal to α-ATP
NADH	Singlet	[0.3, 12] × NAD^+^ intensity (variable)		+0.18 (w.r.t. NAD^+^ center)		Equal to α-ATP
UDPG	Pseudo-Doublet	1:1	[−9.95, −9.60] (upfield line)	+1.6 (w.r.t. upfield line)	[25, 45]	
GPC	Singlet		[+2.90, +3.00]		[7, 50]	
GPE	Singlet		[+3.48, +3.56]			Equal to GPC
PC	Singlet		[+6.18, +6.28]			Equal to GPC
PE	Singlet		[+6.74, +6.82]			Equal to GPC
P_i_	Singlet		[+4.70, +5.00]		[7, 60]	
eP_i_	Singlet	[0.05, 0.75] × P_i_ intensity (variable)		[+0.10, +0.50] (variable w.r.t. P_i_)		[0.05, 2.00] × P_i_ damping (variable)
MPL	Singlet		[+2.15, +2.25]		[15, 35]	
DPG	Pseudo-Doublet	1:1	[+6.00, +6.30] (upfield line)	+0.40 (w.r.t. upfield line)	[30, 80]	

*Note that the constraints for the individual resonance frequencies are adapted in each voxel individually to the local B_0_ estimated in the prefitting step. The frequency shift constraints apply to resonances modeled as multiplets. The reference to which those constraints are referring to are mentioned in brackets. Within a multiplet, equal dampings were constrained to each resonance*.

* *A fixed J-coupling of 17 Hz was assumed for ATP*.

Applied to the processed MRSI datasets, the main quantification step yielded 3D intensity maps (in [a.u.]) for the individual ^31^P resonances of the extended model. For the case of multiplet resonances, e.g. α-ATP, as well as for the pseudo-multiplets, the presented metabolite maps are given as the sum of the individual intensities within the multiplet. In the specific case of NAD(H), the sum of its constituents was calculated: NAD(H) = NAD^+^ + NADH. To enable the comparison to ^31^P quantification models not explicitly accounting for UDPG, eP_i_, and DPG [e.g. at lower fields, (5)], the following surrogate intensity maps were emulated: tNADH = NAD(H) + UDPG/2, tP_i_ = eP_i_ + P_i_, tPC = PC + DPG/2, and tPE = PE + DPG/2. For convenience, it was assumed that DPG intensities are distributed equally across PMEs. Additionally, maps of the eP_i_-to-P_i_ ratio were generated for the interpretation of presented tP_i_ intensities, as well as ratio maps for PMEs [(t)PC, (t)PE] and phosphodiesters (PDE; GPC, GPE), i.e., PC-to-PE, GPC-to-GPE, and GPC-to-PE, suggesting to discriminate the IDH mutation status according to the findings of Esmaeli et al. (7). Finally, to compensate for the intensity scaling induced *via* the coil sensitivity profile, and to improve the comparability of metabolite intensities between subjects, ratio maps of the individual metabolite intensities relative to the local α-ATP intensity were calculated (except for α-ATP itself, which is given subject to the coil sensitivity profile throughout this study).

Note that the quantified metabolite intensities were not transformed to concentration values due to the unknown spatial variation in the required physical quantities, i.e., local T1 times for the individual metabolites and local B1 values.

### Region-of-Interest Analysis

Regions-of-interest (ROI) were defined and processed in the Medical Imaging Interaction Toolkit (MITK) (24) using the acquired morphological ^1^H images.

3D ROIs of tumor tissue were defined on the 3T clinical MRI data co-registered to the morphological 7T images. For the data acquired in patients with high-grade glioma (HGG; CNS WHO grades 3 and 4), the whole tumor volume (WTV) was segmented into central necrosis (NEC), gadolinium contrast enhancement (GDCE), and peritumoral edema (ED). In case of the patients with low-grade glioma (LGG; CNS WHO grade 2), only the WTV ROI was segmented, defined as the volume of the T2-FLAIR hyperintense signal alteration. In addition, healthy tissue ROIs were defined manually on the morphological 7T images in WM (in-between the frontal and parietal lobe) and a region with high content of GM (in-between the parietal and the occipital lobe) in all subjects, with care taken to define those ROIs always in equivalent regions (representative ROI locations are illustrated in Supplementary Figure 1). Note that the WM and GM ROIs in the patients were always defined on the contralateral side at a distance of more than 2 cm from the tumor ROIs, to minimize a potential signal contamination. In case of the healthy subjects, WM and GM ROIs were defined in both hemispheres.

The ROIs defined on the morphology were then mapped onto the ^31^P MRSI grid using a linear interpolation algorithm in MITK (see Figures 1A–C for examples). This resulted in mapped WTV ROI sizes from 109 to 422 voxels in the individual patients (effective volumes from 27 to 103 ml), whereas the mapped WM and GM ROI sizes typically consisted of approximately 10 voxels (Table 2). Furthermore, a mask defining brain parenchyma was also mapped onto the MRSI grid and applied to the 3D ^31^P metabolic maps in order to generate overlays with morphology, showing only voxels in the brain.

**Table 2:** ROI sizes for the individual subjects of the study cohort.

**Subject**	**WM left**	**WM right**	**GM left**	**GM right**	**WTV**	**NEC**	**GDCE**	**ED**
V1	15	12	14	14				
V2	11	10	12	13				
V3	6	7	10	9				
LGG1		10		9	109			
LGG2	7		8		201			
HGG1	10		12		316	3	70	244
HGG2	9		14		422	38	126	266
HGG3		8		12	252	48	153	53
HGG4	9		12		339	48	90	204
HGG5		8		8	246	69	173	13

*Given are the number of voxels within a region-of-interest (ROI) after mapping onto the phosphorus magnetic resonance spectroscopic imaging (^31^P MRSI) grid. White matter (WM) and gray matter (GM) ROIs were defined on both hemispheres for the volunteers (V1–V3), but only on the contralateral hemisphere for the patients (LGG1–HGG5). For the patients with low-grade glioma (LGG1 and LGG2), only the whole tumor volume (WTV) ROI was segmented. Note that in the case of patients with high-grade glioma (HGG1–HGG5), the number of voxels within the mapped WTV ROIs (defined as union of the individual segments on morphology) is not necessarily the sum of voxels in the separately mapped necrosis (NEC), gadolinium contrast enhancement (GDCE) and edema (ED) ROIs, because of voxel assignments to multiple ROIs by the mapping algorithm in some cases*.

The analysis of regional differences in metabolite intensities (w.r.t. α-ATP) and ratios in each subject was performed in MATLAB and is reported as mean and standard deviation within each ROI. For the group analysis, six groups were defined and included the following numbers of ROI means: thirteen for each the WM and GM groups (six ROIs from healthy subjects (both hemispheres) + seven contralateral ROIs from patients), two for the WTV group of the patients with LGG, four for the WTV, four for the ED, and five for the combined GDCE/NEC (C+N) groups of the patients with HGG. The exclusion of the ROI means one of the patients with HGG from the WTV and ED groups based on the fact that in this patient no well-separated edema voxels were present (HGG5 in Table 2).

## Results

### Qualitative Analysis of Localized ^31^P Spectra

In all subjects, localized ^31^P spectra of high quality were obtained throughout the whole brain. Figure 1 shows representative ^31^P spectra from a patient with a LGG (astrocytoma WHO 2) and another patient with a HGG (glioblastoma). Besides the typical signals observed in brain parenchyma arising from higher-concentrated metabolites, like PCr, ATP, GPC, GPE, P_i_, and PE (Figures 1D,E), also resonances from lower-concentrated metabolites were detectable, i.e., eP_i_ in the frequency range between 5.0 and 5.4 ppm (Figures 1G,H), and a resonance dominated by NAD^+^ and NADH around δ ≈ −8.3 ppm (Figures 1J,K). Moreover, in the HGG spectra (Figures 1F,I,L) a broad resonance (typical FWHM ≈ 35 Hz) around δ ≈ 2.2 ppm became prominent, assigned to MPL, as well as a broad resonance (typical FWHM ≈ 25–30 Hz) in the frequency range around δ ≈ −9.8 ppm, assigned to UDPG. Note the broadened baseline underneath the PME resonances, typically manifesting as a shoulder upfield from PC in healthy tissues (indicated by the gray arrow in Figure 1G). This suggests the presence of at least one additional signal component contributing to the PME resonances, which was tentatively assigned herein to DPG.

While the spectra in the tumor region of the patients with LGGs showed only minor differences to the spectra of healthy tissues (cf. Figures 1D,E), pronounced differences to healthy tissue could be observed in the tumor spectra of the patients with HGGs (cf. Figures 1D,F). There, typically a reduction of PCr, ATP, and PDEs, an increase in PC, MPL, and UDPG, as well as a broadening of both increased inorganic phosphate resonances was observed compared to healthy tissue.

### Metabolite Mapping

In general, the extended quantification model enabled robust and reliable quantification of all 12 metabolites observed in the brain parenchyma (Figures 1D–L). In particular, the inclusion of the DPG signal into modeling combined with the constraint of equal dampings on the PME and PDE resonances improved robustness of modeling and enabled extraction of at least parts of the background underlying PE and PC (Figures 1G–I). Moreover, the presence of a detectable UDPG resonance at δ ≈ −9.8 ppm implied to include another UDPG contribution also into the modeling of resonances at δ ≈ −8.2 ppm (Figure 1L). Application of this quantification model to the MRSI datasets yielded 3D ^31^P metabolite maps of high spatial resolution in all subjects.

**Figure 2:**
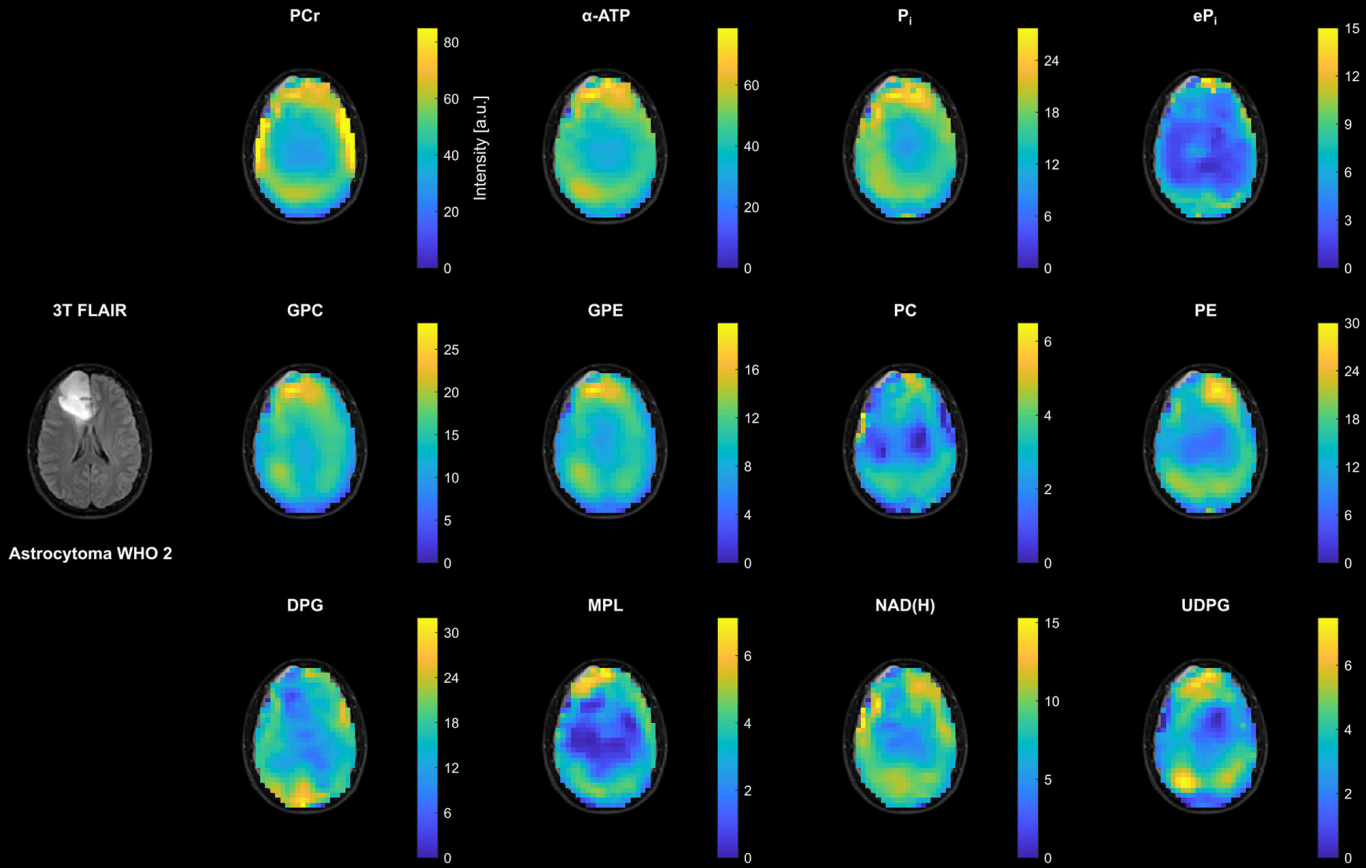
Representative transversal slices of all quantified 3D metabolite maps from the patient with low-grade glioma (astrocytoma WHO 2; cf. Figures 1A,B), shown as intensities in arbitrary units. Note the different scales for the individual metabolites, and that all maps are subject to the coil sensitivity profile. For visual guidance, the metabolite maps are masked to show only brain parenchyma as displayed on the 3T T2-weighted FLAIR images (co-registered to the MPRAGE images obtained at 7T).

Figure 2 shows representative transversal intensity maps of all 12 quantified metabolites in the patient with LGG (astrocytoma WHO 2 from Figures 1A,B). In addition to existing differences between WM and GM, there was an increase in metabolite intensities toward the periphery compared to central brain regions due to the coil sensitivity profile. Despite their low concentration, eP_i_, NAD(H), and in particular UDPG could be quantified reliably in peripheral voxels of healthy tissue, as well as in most central brain voxels. Extraction of the background signal in the PME region, i.e. DPG, was also possible throughout the brain. Although a prominent MPL resonance was not observed in LGG or healthy tissues, maps could be extracted that represent a quantification of parts of the spectral baseline around 2 ppm. In coherence with the spectra in Figures 1D,E, the intensity changes in the LGG tumor region compared to the surrounding healthy tissue were rather weak. The most notable changes were a reduction in DPG, NAD(H) and PE, and a slight increase in UDPG, while the other metabolite intensities appeared to remain unaffected (for comparison, metabolite maps from a healthy subject are shown in the Supplementary Figure 2).

**Figure 3:**
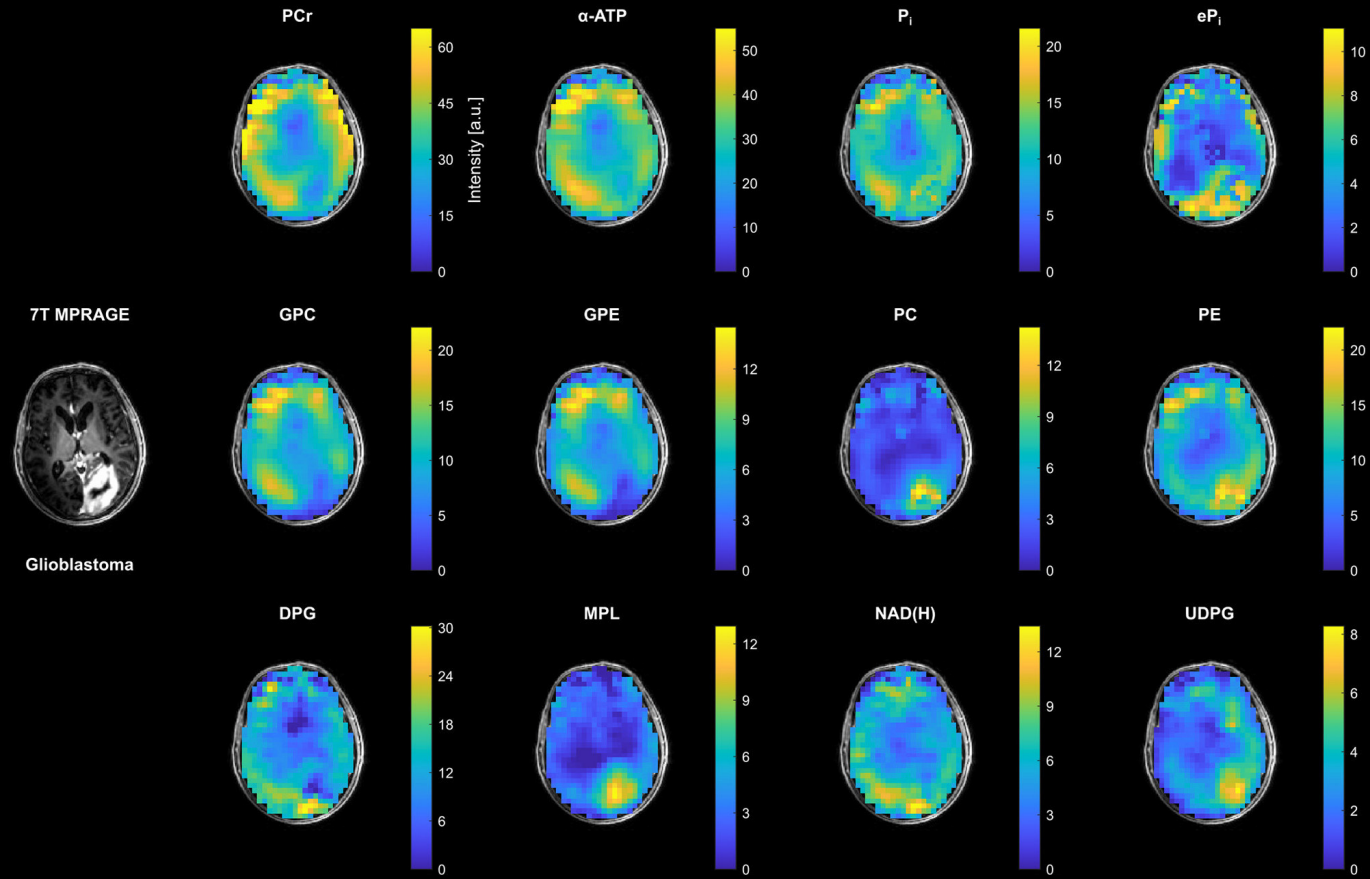
Representative transversal slices of all quantified 3D metabolite maps from the patient with high-grade glioma (glioblastoma; cf. Figure 1C), shown as intensities in arbitrary units. Note the different scales for the individual metabolites, and that all maps are subject to the coil sensitivity profile. For visual guidance, the metabolite maps are masked to show only brain parenchyma as displayed on the 7T non-enhanced T1-weighted MPRAGE images.

In contrast to the small changes in the patient with LGG, clear changes in most metabolite intensities could be observed in the tumor region of the patient with HGG (glioblastoma from Figure 1C) compared to the surrounding healthy tissue (Figure 3). This involves an increase in inorganic phosphates (predominantly eP_i_), PC, PE and UDPG, and a reduction of PCr, ATP, GPC and GPE. The strong increase in MPL intensity in this case was related to the appearance of a well-resolved MPL resonance in the tumor spectra (cf. Figure 1I).

**Figure 4:**
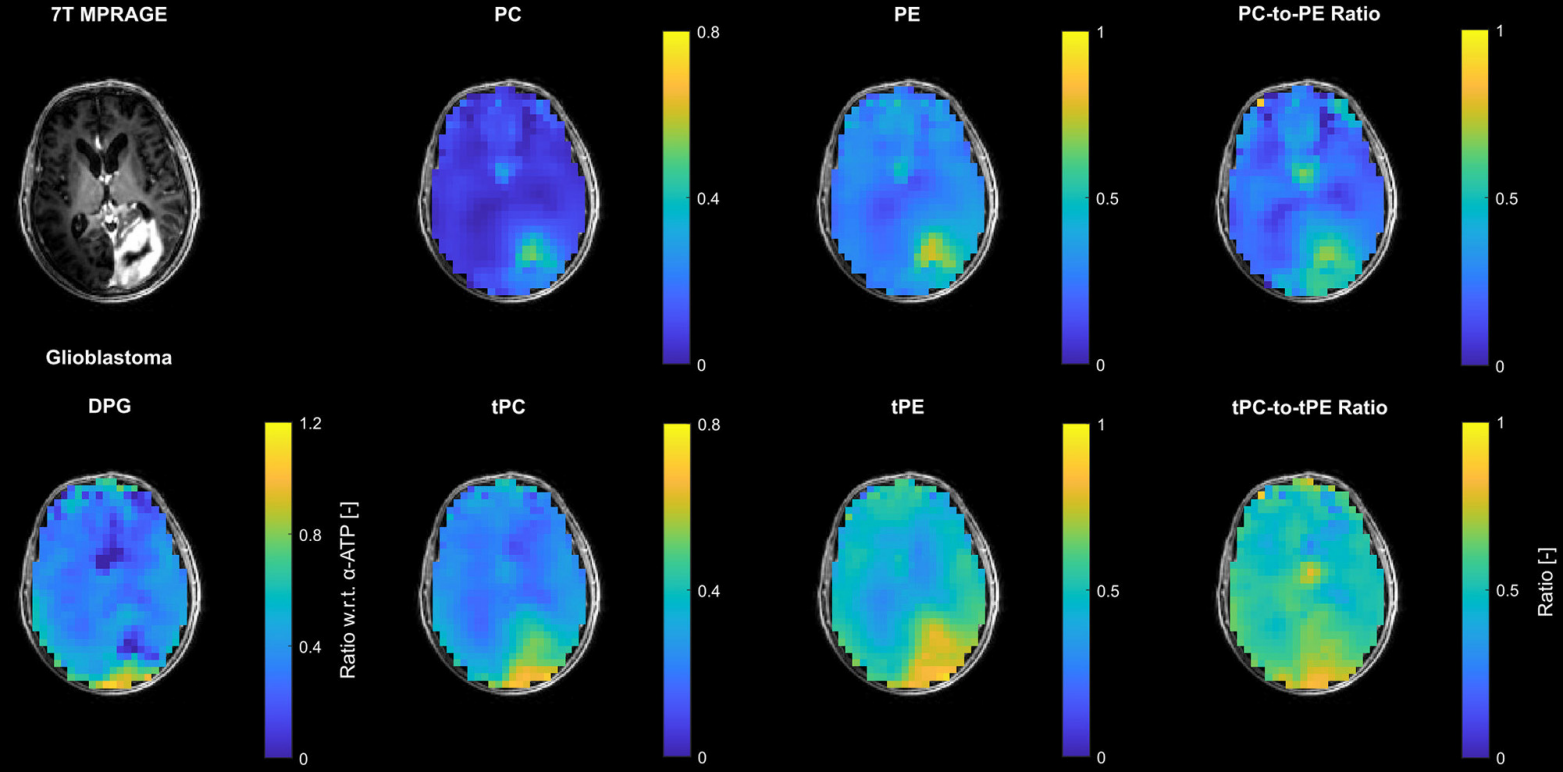
Representative transversal slices of 3D metabolite maps quantified in the PME region from the patient with high-grade glioma (glioblastoma; cf. Figure 1C), shown as ratios with respect to the α-ATP intensity. Note that the scales for the actually modeled (PC, PE) and the corresponding surrogate intensities (tPC, tPE) are the same. For visual guidance, the metabolite maps are masked to show only brain parenchyma as displayed on the 7T non-enhanced T1-weighted MPRAGE images.

Figure 4 shows examples of ratio maps with respect to α-ATP for PC, PE and DPG, and the constructed surrogate intensities tPC and tPE, as well as the corresponding PME ratio maps for the patient with HGG (glioblastoma). These ratio maps are intrinsically compensated for the coil sensitivity scaling, and were used for the ROI analysis (further ratio maps for the individual metabolites are shown in the Supplementary Figures 3–5, corresponding to Supplementary Figure 2 and Figures 2, 3, respectively). Compared to conventional modeling with only two resonances, i.e. tPC and tPE, the reduced PC and PE intensities obtained from the extended quantification model affected also the calculated PME ratio, leading to a different tumor contrast in the PC-to-PE ratio map than in the tPC-to-tPE ratio map.

### ROI Analysis

**Figure 5:**
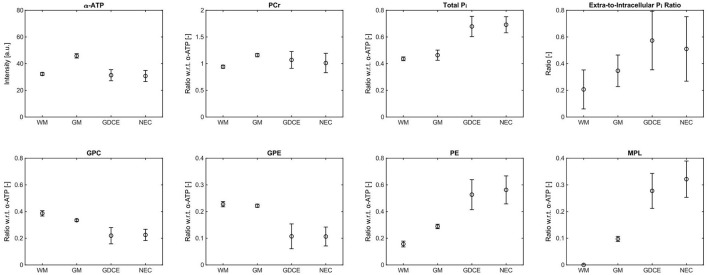
Representative ROI analysis of selected metabolite intensities from the patient with high-grade glioma (glioblastoma) shown in Figures 3, 4, given as mean (circles) ± standard deviation (bars). Only the α-ATP intensities are given in arbitrary units, and subject to the coil sensitivity profile; all other metabolites are quantified as ratios with respect to the α-ATP intensity. Number of voxels *n* in each ROI: *n*_WM_ = 8, *n*_GM_ = 8, *n*_GDCE_ = 173, *n*_NEC_ = 69.

The high spatial resolution of the metabolite maps not only enabled to assess differences between healthy and tumor tissues, but also between individual diseased tissue compartments. Figure 5 shows a representative ROI analysis of selected metabolite intensities for the patient with HGG shown in Figures 3, 4. Since in this patient no well-separated edema voxels were present, only four ROIs were considered here, i.e., contralateral WM and GM, GDCE, and NEC. Furthermore, this patient showed most of the extreme values in tumor ROIs. Comparing the means of the diseased to the healthy tissue ROIs, there was a clear difference observable in most metabolite intensities. However, no clear difference could be observed between the GDCE and NEC ROIs, taking into account the larger standard deviation in the diseased tissue compartments, which is related to the large extent of the ROIs. In all the other patients with HGG, no clear difference between the GDCE and NEC ROIs was observed either, leading to the conclusion to merge those ROIs (C+N) in the following analysis. Note that in the other patients with HGG, where well-separated edema voxels were present, a difference between the GDCE/NEC and the ED ROIs was suggested (see group analysis below). The Supplementary Tables 6–17 in the Supplementary Material summarize the ROI results for the individual metabolite intensities of the whole study cohort.

**Figure 6:**
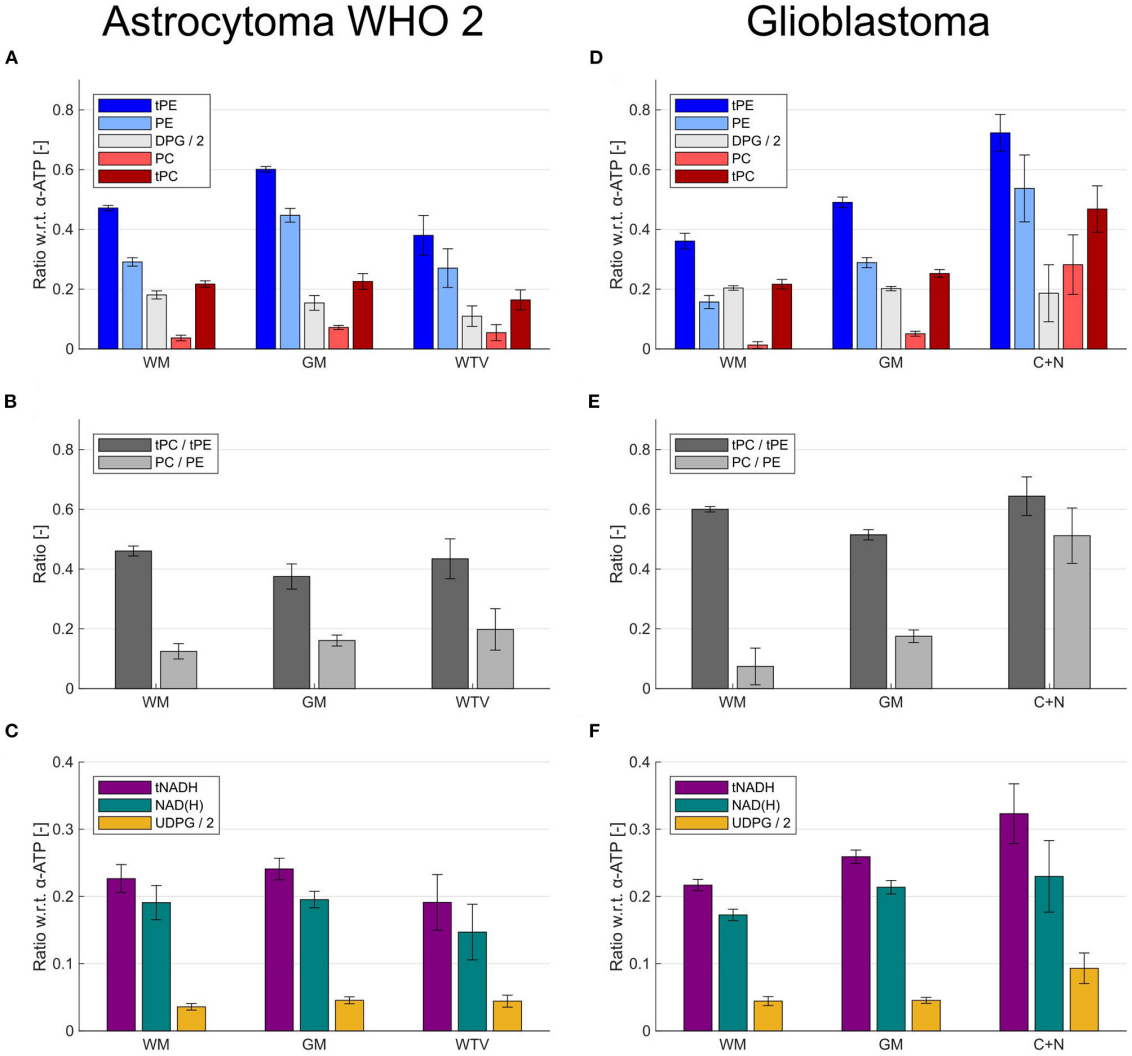
Bar plots illustrating the effects of the extended modeling on the quantification results within different ROIs (healthy and tumor tissues) from the patient with low-grade glioma (astrocytoma WHO 2) shown in Figure 2 **(A–C)** and the patient with high-grade glioma (glioblastoma) shown in Figures 3 and 4 **(D–F)**. **(A,D)** Effect of DPG inclusion on the quantified intensities of PC and PE. **(B,E)** Effect of DPG inclusion on the ratios derived from PC and PE. **(C,F)** Effect of UDPG inclusion on the quantified intensities of NAD(H). The metabolites are quantified as ratios with respect to the α-ATP intensity.

Figure 6 illustrates effects of the extended quantification model on the ROI results for the patient with LGG (astrocytoma WHO 2; Figures 6A–C) and the patient with HGG (glioblastoma; Figures 6D–F). Although the inclusion of DPG into modeling introduced simply an offset to the individual intensities of PC and PE (Figures 6A,D), it strongly affected the derived PME ratios used as tumor markers in the individual ROIs (Figures 6B,E). This can be seen best in the patient with HGG (Figure 6E), where the conventional tPC-to-tPE ratio is reduced by 14% in GM compared to WM, whereas conversely the PC-to-PE ratio is increased by 136%. The change in contrast is even more drastic in the tumor, where the conventional tPC-to-tPE ratio increases only marginally by 7% compared to WM, but the PC-to-PE ratio increases strongly by 592% (cf. Figure 4). This increase in contrast due to the inclusion of DPG could be beneficial in situations with more subtle changes, as in the case of the patient with LGG (Figure 6B), where an increase of the PC-to-PE ratio of 59% was observed in the tumor compared with WM. Furthermore, the extended quantification model facilitated the interpretation of intensity changes observed for tNADH by inclusion of the UDPG resonance. While the trend to decreased tNADH intensity by 21% in the patient with LGG seemed to be solely associated with a loss of NAD(H) (Figure 6C), the increase in tNADH intensity by 49% in the patient with HGG was associated with a 110% increase in UDPG and 33% increase in NAD(H) intensities (Figure 6F).

From the comparison of the WM and GM ROI mean values for each metabolite shown in Figures 5, 6, it becomes clear that a WM-GM difference is detectable for most metabolites. These WM-GM differences were observed in all subjects, but with varying numerical values for each metabolite and subject (cf. Figures 6A,D and C,F), most likely related to age differences. Note that we found no clear difference between the WM and GM ROI group means in the healthy volunteers compared to the corresponding WM and GM ROI group means in the patients. Therefore, the results of the ROI analysis of the healthy subjects were included in the following group analysis, to increase the range of values considered as normal.

### Group Analysis

**Figure 7:**
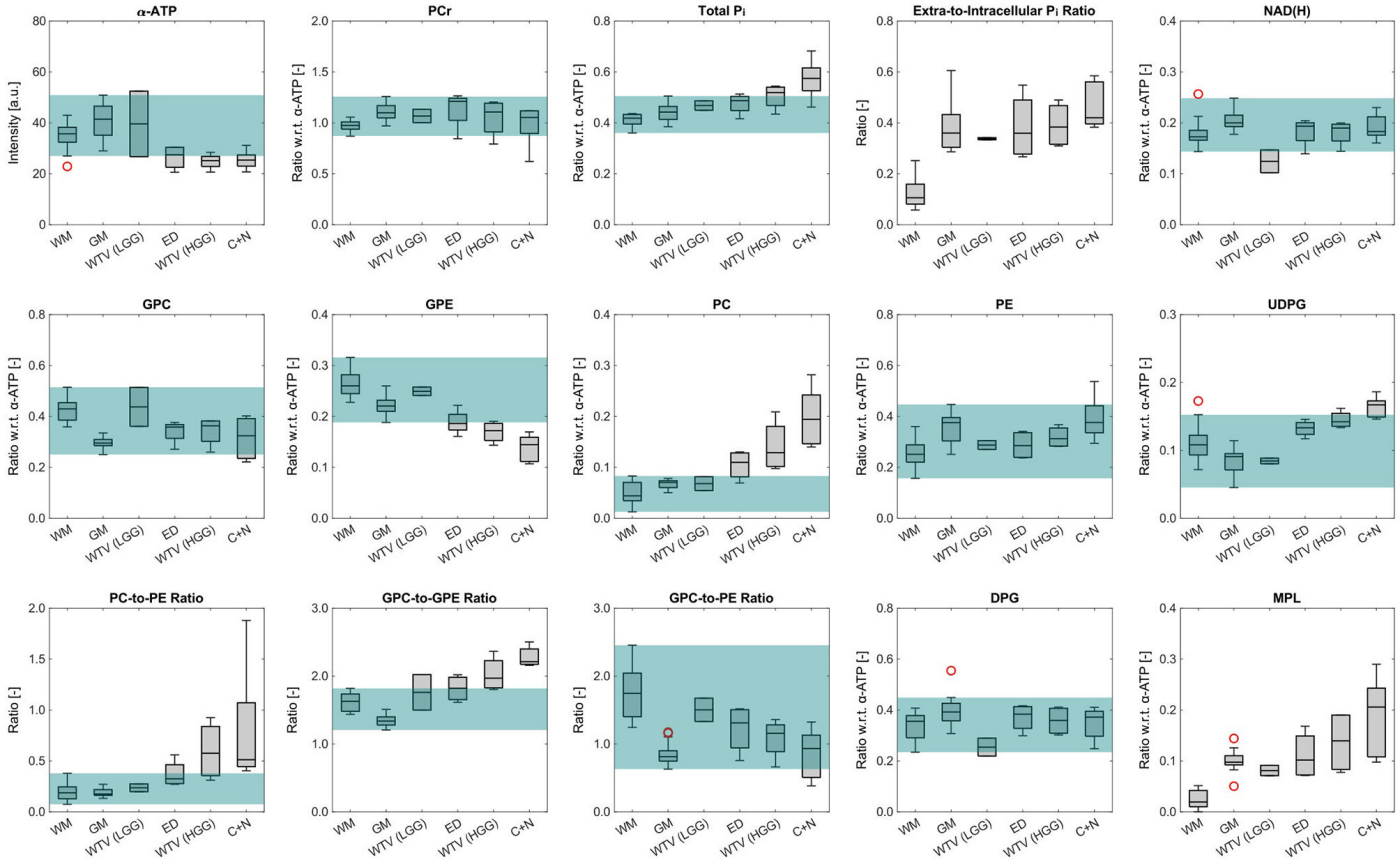
Box plots of the mean metabolite intensities or ratios within the different ROI groups across all subjects investigated in this study. Only the α-ATP intensities are given in arbitrary units, and subject to the coil sensitivity profile; all other metabolites are quantified as ratios with respect to the α-ATP intensity. The green shaded areas indicate the range of values considered to be normal (ignoring outliers in the box plots; red circles). Note that no green shading is shown in case of the extra-to-intracellular P_i_ ratios as no values would exceed the normal range, and in case of the MPL intensities because of the different interpretation of quantification results for the HGG ROIs compared to the other ROIs. Number of ROIs *n* included in each group, as described in detail in the methods section: *n*_WM_ = 13, *n*_GM_ = 13, *n*_WTV_ = 2 (LGG), *n*_ED_ = 4 (HGG), *n*_WTV_ = 4 (HGG), *n*_C+N_ = 5 (HGG).

To identify the most significant metabolic changes in tumor tissues observed across all subjects, box plots of the mean metabolite intensities or ratios within different groups of ROIs are illustrated in Figure 7. A change was considered significant if the corresponding median was exceeding the range of normal values defined by the combined maximum and minimum of WM and GM. The green shaded area in Figure 7 gives a visual guidance for this normal range (ignoring outliers in the box plots; red circles).

With regard to the patients with LGG, the only significant change was observed for NAD(H), with a reduction of the median in the WTV ROI by 28% compared to the median of WM (taken as the reference). In the patients with HGG on the other hand, several significant changes could be observed, with the C+N ROI median deviating strongest from the WM median (given in the following). The most extreme change was observed for PC, increasing by 340%, which also led to a strong change in the PC-to-PE ratio, increasing by 174% (PE is increasing by 50%, but did not exceed the range of normal values). The other extreme increase was given for MPL, which however was not quantifiable since it was typically not detectable in WM. Further significant changes were the increase in UDPG by 54%, an increase by 37% of total P_i_ with a strong shift toward the extracellular component (+298%), and reductions of 45 and 29% for GPE and ATP, respectively. The other metabolite intensities showed only insignificant changes (PCr: +8%, NAD(H): + 6%, GPC: −25%, DPG: +5%). Note that the deviations of the corresponding ED ROI medians from the WM medians were generally smaller and the WTV ROI medians line up in between the ED and C+N medians.

## Discussion

In this study, we applied ^31^P MRSI at an ultra-high *B*_0_ of 7T to a small cohort of patients with glioma to investigate the additional value provided by multiple metabolite maps for clinical research of brain tumors. As with this pilot study we aimed to assess what could be in principle possible at 7T, we decided to apply our recently proposed examination protocol with 51 min acquisition duration for ^31^P MRSI of the human brain (10). By doing so, we obtained spectra of both, high SNR enabling the localized detection of metabolites like NAD(H) and UDPG with low concentrations of only about 0.3 mM (14, 15), and small effective voxel sizes of 5.7 ml enabling a proper separation of individual tissues. From these ^31^P MRSI datasets, 3D intensity maps of 12 identifiable metabolites could be created, demonstrating, to our best knowledge for the first time, the feasibility to extract an extended ^31^P metabolic profile from healthy and diseased brain tissues, as well as their subsegments.

The analysis of these maps revealed the metabolic heterogeneity of the individual tissue types (cf. Figures 2, 3, 7). Although a direct comparison to other studies is not possible because of differences in the experimental setups and investigated cohorts, and the limitations to obtaining absolute metabolite concentrations, some general features are shared across this and earlier studies. In agreement with ^31^P MRSI studies analyzing regional differences of the healthy human brain (25–27), we obtained lower PCr, higher PDE, and lower PME intensities in WM compared to GM. Taking the higher coil sensitivity scaling in GM into account, the sensitivity-corrected ATP intensities in Figures 5, 7 should also be higher in WM, as reported earlier. Moreover, from ^1^H MRSI studies it is known that the total Creatine intensity (tCr; as the sum of creatine and PCr) is lower, and the total Choline intensity (tCho; mainly as the sum of PC and GPC) is higher in WM compared to GM, as observable in high-resolution metabolite maps e.g. in (28). Our results correspond also with these observations, assuming that lower PCr levels are a direct consequence of lower tCr levels, and that GPC is dominating the tCho signal in healthy brain tissue. Regarding the results obtained in ^31^P studies of patients with brain tumors (5, 29, 30), similar findings had been made here for the alterations in phospholipid metabolism, e.g. decreased GPE and increased PC intensities, as well as for the increased intensity of inorganic phosphates tP_i_ in tumor tissue (here the C+N ROI in patients with HGG) compared to healthy tissues. The increase in tP_i_ intensity was dominated by the extracellular contribution, which is in line with our earlier observations (10) (3 of 5 patients with HGG were already presented). In a recently published high-resolution ^1^H MRSI study on a larger cohort of patients with HGG (31), the tCr intensity showed varying trends across tumor subsegments and individual patients, with tCr reductions typically occurring in the necrosis. Our observation of an insignificant change of PCr intensities in tumor tissue across the cohort might correspond to this, despite the clear reduction of the PCr intensity in the central tumor region observed in examples Figures 1F, 3. The same ^1^H MRSI study revealed also heterogeneity across segments and patients for the increase of tumorous tCho intensities, acting as a general diagnostic feature (32, 33). The strong but heterogeneous increase in tumorous PC intensities in our study is in line with this observation, considering the relatively weak and heterogeneous decrease of GPC intensities in tumor tissue. Despite the fact that the ^31^P metabolite intensities obtained in our current study are subject to a tissue-specific T1-weighting (additionally affected by potential spatial variations in flip angles and NOE enhancements), it is reasonable to assume that the observed variations are mainly reflecting concentration changes, since otherwise unrealistic changes in T1 times should have occurred under the applied sequence parameters. To finally prove this and also to improve comparability between studies, however, efficient T1- and B1-mapping techniques for ^31^P MRSI of the human brain would be required enabling the estimation of concentrations.

Also, this study made some findings about the metabolic heterogeneity of brain tumors which had not been described beforehand *via* ^31^P MRSI: compared to healthy tissues, a reduced NAD(H) intensity was observed in LGG tissues, whereas increased UDPG and MPL intensities were observed in HGG tissues. Note that although not proving significant in the group analysis, an increase in eP_i_ intensities was also observed in the spectra of LGG tissues (cf. Figure 1H). Moreover, the extraction of a background signal underlying the PME resonances (tentatively assigned to DPG), led to an increased contrast in PME intensity ratios between healthy and diseased tissues (cf. Figures 4, 6). These novel findings had been made possible *via* the implementation of an optimized quantification model, and the analysis of regional variations in the high-resolution metabolite maps.

### Modeling of an Extended ^31^P Metabolic Profile

Modeling of the extended metabolic profile based on (I) our recently published, highly robust quantification method for ^31^P brain MRSI datasets (10), refined by the inclusion of linear relations and an algorithmic improvement in the prefitting step, and on (II) the analysis of the spectra of healthy and diseased brain tissues, implying also to include resonances for UDPG at δ ≈ −8.2 ppm and δ ≈ −9.8 ppm, and MPL at δ ≈ 2.2 ppm. Moreover, a signal background underlying the PME resonances was included into the extended model, for which earlier ^31^P MRS studies at 7T in the human brain already provided evidence (20, 21), speculating about DPG from blood (34) as a potential contributor. Taking into account that the T2s of the PME and PDE resonances should be of the same magnitude (20), thus also their observed linewidths/dampings, the broadened baseline in the PME region observed in our study (cf. Figure 1G) is in line with those findings, and justifies the inclusion of an additional resonance in this region while constraining equal PME/PDE dampings. Interestingly, without the additional resonance and unconstrained dampings, the quantified linewidth of PC was larger than those of PE, GPC and GPE in healthy tissues, and narrowed in tumor tissues toward the linewidths of PE, GPC and GPE as the PC intensity becomes dominant, further substantiating the applied modeling scheme. The observation that in all subjects the background signal intensity is highest in the back of the head (cf. DPG in Figures 2–4), where the sinuses are located, indeed suggests a relation to deoxygenated blood, forming the rationale for assigning the background mainly to DPG, with modeling parameters estimated from the results of Labotka (22) (deoxygenated blood: δ ≈ 6.0–6.7 ppm). Although it remains unclear which further metabolites contribute to the PME background, not accounting for its increase in those regions would lead to a potential misinterpretation in PME intensities and derived ratios (Figure 4). In this regard, the worse chemical shift dispersion at lower fields bears the risk to miss the background contribution. To clarify the origin of those signals and potentially identify other contributions to the PME background, ^31^P MRSI studies at even higher fields with a better chemical shift dispersion would be helpful, which then could be incorporated also at 7T to further improve modeling.

The parameters for the quantification model described in Table 1 were defined from a thorough preliminary analysis of observed linewidths and frequencies in our study cohort, and enabled reliable modeling of all 12 metabolites throughout the whole brain. The reliability of modeling overlapping resonances was only compromised in voxels with too broad PDE/PME resonances (FWHM > 20 Hz), leading to a potential underestimation of DPG intensities, and thus to increased estimates of PE and PC intensities compared to voxels with clearly separable background. The same applies to voxels with too broad inorganic phosphate resonances (FWHM > 25 Hz for P_i_ and FWHM > 30 Hz for eP_i_), leading to an inability to clearly separate the intensities of the intra- and extracellular compartment. These situations occurred in some of the tumor voxels, and was most probably associated with changes in microscopic susceptibility and a stronger heterogeneity in tissue pH (10). Nevertheless, the quantification of total intensities of overlapping resonances, i.e., tPC, tPE (Figure 4), as well as tP_i_, could be considered as reliable and robust in all cases, offering the possibility to resort to such surrogate intensities, even in the presence of potential ambiguities. Note that although the sum of NAD^+^ and NADH intensities, i.e. NAD(H), could also be considered as reliable and robust, the individual intensities were not due to their still limited SNR, which prevented a robust analysis of the redox potential RX within this study.

Altogether, these observations make the application of the proposed extended quantification model to ^31^P MRSI datasets of the human brain at 7T highly reasonable, and thereby, also provides an additional value for the application to patients. Note that although the focus of this study were the metabolite intensities, the extended quantification model also improves the interpretation of tissue pH estimates by separating the individual inorganic phosphate contributions (10).

### Improving the Interpretability of Quantified Metabolite Intensities

A problem in dealing with the limited spatial resolutions typically accessible with ^31^P MRSI *in vivo* are partial volume effects between different tissue compartments, leading to a mixing and therefore to a dilution of their metabolic differences. The high spatial resolution achieved in this study, however, enabled the clear separation of individual voxels mainly belonging to WM and GM, as well as to tumor tissue. As a consequence, clear metabolic differences between healthy and tumor tissue voxels could be observed in both the spectra (Figure 1) and in the metabolite maps (Figures 2–4). Moreover, the group analysis in Figure 7 also suggests differences between diseased tissue compartments in the HGGs, i.e., edema (ED) and regions with gadolinium contrast-enhancement and necrosis (C+N). This could have been expected as the edema consists of a mixture of healthy and infiltrative tumor cells, which is in line with the linear trends in observed mean metabolite intensities between compartments, as going from healthy tissue ROIs, over the ED ROIs, to the C+N ROIs in Figure 7. This finding has been made possible by the investigation of relatively large tumors in this study, where the smallest compartment size from all ED and C+N ROIs was about 12 ml (belonging to one of the edemas). Note, however, that the spatial resolution is still not high enough to clearly separate the typically small necroses (<25% of WTV) from the larger surrounding contrast-enhancing tissues. This was reflected in the general lack of a measurable difference in metabolite intensities between GDCE and NEC ROIs (cf. Figure 5), making it reasonable to merge both those ROIs into one.

Regarding healthy tissues, our ROI analysis showed a variation in the obtained numerical values for each metabolite and subject, in addition to the general differences between WM and GM (cf. Figures 5, 6). Although this could have been expected for the investigated cohort, e.g., due to age-related changes in brain metabolism (35), or slightly varying WM/GM fractions (25, 26) across the ROIs in each subject, this increases the range of metabolite intensities that could be considered as physiologic. This variability complicates the identification of metabolic changes related to tumor tissue when they are subtle compared to changes in different healthy tissues, as it presumably is the case for LGGs. Further note that in the presence of residual partial volume effects with healthy tissue, a large extent of a tumor ROI expanding over different brain regions contributes to a large standard deviation.

This situation complicates even more in the case of lower spatial resolution. To illustrate the effect for the case of the HGGs, metabolite intensities averaged over all tumor subsegments were additionally calculated for the individual patients, resulting in the WTV(HGG) values of Figure 7. Here, the pronounced metabolic differences from the C+N ROI are diluted, depending on the portion of edema, leading to a potential loss of a difference compared to healthy control tissue upon WTV averaging. This poses a challenge particularly for small tumors compared to the effective voxel volume, where additionally partial volume effects with healthy tissue compartments further obscure potential subtle differences.

Taken together, the final metabolite intensities in tumor tissue will depend on the anatomical region, the surrounding WM/GM tissue fractions mixing in, as well as the substructure of tumor tissue, thus complicating interpretation and comparison to a potentially not well-defined reference tissue. The higher spatial resolution available for ^31^P MRSI at 7T provide an added value to the interpretation of data obtained from brain tumors, as it allows to select spectra belonging more specifically to a certain tissue type, being important to analyze subtle metabolic differences. This improvement in specificity might become of increasing importance in the future, as a recent study (30) suggests that subtle metabolic differences of gliomas detectable *via* ^31^P MRSI might extend further than visible on morphological images. That such a metabolic heterogeneity in the peritumoral region is indeed detectable was recently demonstrated by high-resolution ^1^H MRSI (31).

To account for the observed variability, we defined the range of values considered to be normal for our study cohort (green shaded areas in Figure 7), and identified those metabolite intensities as particularly interesting markers indicating tumor tissue that exceeded this large range within the group. Note, however, that ROI-specific changes in each subject might be more or less pronounced. According to that paradigm, these markers were NAD(H) for the group of LGGs, and tP_i_, GPE, PC, MPL, and UDPG for the group of HGGs. Note that in case of the reduction of ATP in the HGG group, a variation in intensity due to coil sensitivity profile must further be taken into account. Further note that the anticipated reduction of mean PCr intensity in HGGs could have been possibly weakened due to residual signal bleed from the dominant PCr signal from neighboring muscle tissues (either neck or temporal muscles), being typically too close to the borders of the tumor ROIs.

### Novel Insights Into Tumor Biochemistry

The alterations detected in the intensities of NAD(H), UDPG, MPL, and PC (after extraction of background signals) have the potential to provide novel insights into the biochemistry of gliomas *in vivo*. Note, however, that the small number of subjects investigated in this pilot study limits the significance of the observations made and prohibits a reasonable statistical analysis, also considering the large variance in biochemistry of the individual glioma subtypes. Thus, the presented results should be understood as a guidance for future research. To draw definite conclusions on glioma biochemistry, studies on larger patient cohorts of the individual glioma subtypes will be necessary.

Besides its potential to assess the redox potential in tumor tissue, the NAD(H) intensity reflects the general availability of redox equivalents for energy generation. In our cohort, the mean NAD(H) intensities were reduced in both patients with LGGs, bearing mutant IDH1, while the patients with HGGs (4 of 5 with wildtype IDH) appeared to show normal values of NAD(H) (Figure 7). Interestingly, the only patient with HGG also bearing mutant IDH1 (astrocytoma, CNS WHO grade 3) had the lowest mean NAD(H) intensity, suggesting a relation between the NAD(H) level in tumor tissue and the IDH mutation status. That this could be indeed the case, is substantiated by a recent report showing in a glioma model that mutant IDH1 is associated with reduced levels of NAD(H), compared to the levels in IDH wildtypes (36). Contrary to this, UDPG intensities were increased in the patients with HGGs, while the patients with LGGs appeared to show normal values. As UDPG species are heavily involved in the glycosylation of biomolecules, which e.g. affects the function of proteins and membranes manifold, this increase in aggressive tumors could have several interpretations and remains to be investigated in the future. A recent study using ^1^H MRS showed an increase in some UDPG species in glioma cells as a response to cisplatin treatment (37), thus, rendering UDPG detection particularly interesting to assess treatment response. Note that due to the more challenging detection of the UDPG resonance at δ ≈ −9.8 ppm at lower fields, possibly only the tNADH intensity at δ ≈ −8.3 ppm is assessable in this case, which might lead to a potential misinterpretation of its intensity changes (cf. Figures 6C,F). In this regard, ^31^P MRSI at 7T may provide an additional value in the application to gliomas as it enables the clear separation of NAD(H) and UDPG intensities.

Another interesting finding is the strong increase of MPL intensity in the patients with HGGs (Figures 1F,I). The broad baseline in this spectral region that is typically detectable in healthy brain tissue originates from phospholipids in macromolecular structures mobile enough to be NMR-visible (38), and was partially extracted *via* the quantification model also in this study (cf. MPL maps in Figures 2, 3). Other than that, the increased signal of narrowed linewidth that is observed in tumor tissue is likely to originate from even more mobile phospholipids in smaller vesicular structures, and could be associated with necrosis (cf. Figure 5) as a result of degradation processes. A recent finding of ^31^P MRS at 7T in fibroglandular breast tissue (39) attributed the MPL resonance at 2.2 ppm with similar characteristics more specifically to Glycerophosphatidylcholine in small vesicles, which might possibly be the correct assignment in our study too. Due to the reduced baseline intensity at 7T as a consequence of stronger relaxation *via* chemical shift anisotropy compared to lower field strengths, the detectability of the tumor MPL resonance might be enhanced, thus representing a selective marker for malignant tissues, which would provide a further added value.

With respect to the strong increase in PC and the reduction of GPE intensities in HGGs, it is well-known that the metabolic alterations in tumors lead to enhanced membrane turnover in order to promote cell proliferation. This leads to the rationale to use an increased PME-to-PDE ratio (i.e., PE-to-GPE and PC-to-GPC) compared to healthy tissues as a marker indicating malignant tissue. In accordance with this, we observed increased PME and reduced PDE intensities in patients with aggressive HGGs (Figures 4, 5, 6D, 7), leading to increased PE-to-GPE and PC-to-GPC ratios (data not shown, but can be easily inferred). However, the provided evidence regarding a background underlying the PME resonances (e.g., DPG in Figure 1G) potentially impacts interpretation, as it reduces PME intensities, thus also altering the ratios that should indicate tumor tissue (cf. Figure 4). Indeed, the inclusion of a background signal leads to more drastic changes in PC intensities in tumor compared to healthy tissues (Figures 6D, 7), being more in line with concentration changes obtained from metabolomics studies (40, 41) particularly for higher-grade tumors.

In this study, we decided to focus on the effects of including a baseline into modeling for the ratios introduced by Esmaeli et al. (7), i.e., the PC-to-PE, GPC-to-GPE, and GPC-to-PE ratios, as their results on glioma xenografts in tissue extracts and *in vivo* demonstrated the potential to discriminate wildtype and mutant IDH species. A recent clinical study of ^31^P MRSI in patients with glioma at 3T (9) could not validate this finding, attributing this to potential limitations in sample size and spatial resolution. Indeed, by using the conventionally modeled tPC-to-tPE ratio in the examples shown in Figures 4, 6B,E, the difference between ratios in tumor tissues is not standing out against healthy tissues, and would likely be further diluted in scans with lower spatial resolution. Using instead the PC-to-PE ratio led to an increase in contrast against healthy tissues in these specific cases, and showed lower values in mutant-IDH tumors (both LGGs and the HGG with WHO grade 3) compared to wildtype-IDH tumors (glioblastomas in the HGG group) in Figure 7, thus hinting at a potential discrimination of IDH species [however, with reversed ratios compared to (7)]. Regarding the GPC-to-GPE and GPC-to-PE ratios, trends between species could also be suggested by our results. Note, however, that given the MPL resonance observed in patients with HGGs originates from Glycerophosphatidylcholine, it is reasonable to assume also the presence of Glycerophosphatidylethanolamine in the spectra, which overlaps with GPC (39) and thereby might obscure the true ratios involving the GPC intensity. Nevertheless, further studies on larger patient cohorts are necessary to validate the observed changes in the PME/PDE region and to enable their proper interpretation. In this regard, ^31^P MRSI at 7T may provide another added value.

### Toward Clinical Applicability

The high quality of the data obtained in this study resulted to a non-negligible contribution from the SNR gain at higher fields. However, taking the step toward higher *B*_0_ is not replacing the need for further improvements in SNR, as by using dedicated hardware like a receiver array, optimized sequence parameters, and ^31^P-{^1^H}-NOE enhancements, to foster the applicability of ^31^P MRSI in patient studies.

In this regard, further improvements can be achieved *via* optimized data processing techniques. Besides the application of an optimized quantification model, another important contributor was the application of low-rank denoising techniques (42) to extract the small signals of low-concentrated metabolites from voxels of small size. To preserve the spectral features of the low-intensity signals with reduced bias, i.e. actual resonance frequencies and linewidths, we employed the *Moderate Low-Rank Denoising* approach described in (10) with the same denoising parameters. This was reasonable since the requirements for denoising regarding the required SNR gain for a reliable quantification of low-intensity signals were comparable across the relevant metabolites having similar sensitivities, i.e, UDPG, NAD(H), and eP_i_.

Due to the high number of signal averages acquired in the herein utilized MRSI protocol, which results in a long measurement duration, practicability for clinical research is currently not yet given. However, we recently demonstrated that low-rank denoising also provides the opportunity to reduce the measurement duration in high-resolution ^31^P MRSI acquisitions, by simply substituting the acquisition of additional signal averages by a more aggressive denoising scheme (10, 43). As outlined earlier, this strategy requires careful consideration not to bias final quantification results, with its reliability depending on the SNR of the ^31^P MRSI acquisition. In this regard, the SNR gain at higher *B*_0_ provides a benefit for denoising, and additionally the application of different sequence parameters may help to optimize SNR on specific metabolites. An alternative approach in this way to speed up the MRSI protocol would be the application of SPICE techniques (44, 45).

Although the detailed analysis of such strategies is beyond the scope of this paper, and must be investigated in future studies, some coarse prospects can already be given here: shortening the measurement time to 20 min for the given acquisition and processing scheme (only about 40% of signal averages) would reduce the SNR by about 35%, which is well-tolerable for the high-intensity resonances like PCr, ATP, PDEs, PE, and P_i_ presumably without compromises in quality, and potentially also for resonances with strongly increased intensities in tumor tissues, like PC or MPL. For those resonances, even a shorter measurement duration would be conceivable while maintaining the spatial resolution. For the low-intensity resonances like NAD(H) and UDPG, the reduction in SNR would have to be compensated by a reduction in spatial resolution to at least (1.5 cm)3 nominal voxel volume (effective voxel volume of about 10 ml) to obtain approximately the same data quality as shown in this study. In these ways, measurement time could be reduced to clinically more relevant time scales while still maintaining a reasonable spatial resolution for separating individual tissues and a reliable quantification, e.g., on the order of 10 ml voxels in 20 min, fostering the applicability of ^31^P MRSI in patients with glioma at 7T.

## Conclusion

Altogether, this pilot study on a small patient cohort demonstrates that ^31^P MRSI at 7T has the potential to add value to the clinical research of glioma. The sensitivity and spectral resolution available at UHF in combination with an optimized quantification model enables the reliable mapping of an extended ^31^P metabolic profile in patients that could provide novel insights into tumor biochemistry *in vivo*. This includes low-concentrated metabolites like NAD(H) and UDPG, which might generate information of diagnostic and therapeutic relevance, as well as improved estimates for PMEs, which might affect metabolite ratios typically used as tumor markers in a beneficial way. Moreover, the attainable small effective voxel sizes on the order of 10 ml enable a meaningful separation of spectra from different tissue types, like cerebral WM and GM, as well as subsegments in larger-sized tumors. This improves the interpretability of ^31^P metabolite intensities obtained from malignant tissues—in particular when only subtle differences compared to healthy tissues are expected as it presumably is the case for LGGs—that being potentially diluted by partial volume effects with healthy tissues showing a large physiologic variability in metabolite intensities. Although the applied MRSI protocol of long duration is currently of limited practical use, we pointed out that further optimization of data acquisition and processing techniques will bring the anticipated value easily into the reach of clinically applicable timeframes on the order of 20 min. With this, the required studies on larger cohorts of patients with brain tumors will become feasible, which will finally enable to assess the full clinical value of this technique.

## Data Availability Statement

The raw data supporting the conclusions of this article will be made available by the authors, without undue reservation.

## Ethics Statement

The studies involving human participants were reviewed and approved by Ethics Committee of the Medical Faculty of the University of Heidelberg. The patients/participants provided their written informed consent to participate in this study.

## Author Contributions

AK, NW, and DP contributed to the conception and design of the study and to the data acquisition. AK, NW, VF, JB, SG, and DP performed the data analysis. AK, VF, JB, and SG contributed to the method development. H-PS, ML, and PB coordinated the project. DP supervised the study. AK wrote the first draft of the manuscript. All authors contributed to critical manuscript revision, read, and approved the submitted version.

## Conflict of Interest

The authors declare that the research was conducted in the absence of any commercial or financial relationships that could be construed as a potential conflict of interest.

## Publisher's Note

All claims expressed in this article are solely those of the authors and do not necessarily represent those of their affiliated organizations, or those of the publisher, the editors and the reviewers. Any product that may be evaluated in this article, or claim that may be made by its manufacturer, is not guaranteed or endorsed by the publisher.

## Abbreviations

ATP: Adenosine-5′-Triphosphate
CNS: central nervous system
C+N: gadolinium contrast enhancement + necrosis
DPG: 2,3-Diphosphoglycerate
ED: edema
eP_i_: extracellular inorganic phosphate
GDCE: gadolinium contrast enhancement
GM: gray matter
GPC: Glycerophosphocholine
GPE: Glycerophosphoethanolamine
HGG: high-grade glioma
IDH: isocitrate dehydrogenase
LGG: low-grade glioma
MITK: Medical Imaging Interaction Toolkit
MPL: mobile phospholipids
MRSI: magnetic resonance spectroscopic imaging
NAD(H): Nicotinamide Adenine Dinucleotide
NEC: necrosis
NOE: nuclear Overhauser effect
PC: Phosphocholine
PCr: Phosphocreatine
PDE: phosphodiesters
PE: Phosphoethanolamine
P_i_: intracellular inorganic phosphate
PME: phosphomonoester
ROI: region-of-interest
SNR: signal-to-noise ratio
tCho: total Choline
tCr: total Creatine
UDPG: Uridine Diphosphoglucose
UHF: ultra-high field
WHO: World Health Organization
WM: white matter
WTV: whole tumor volume.
