# Supplementary material for: Mapping an Extended Metabolic Profile of Gliomas Using High-Resolution 31P MRSI at 7T
Source: Front Neurol. 2021 Dec 23;12:735071. doi: 10.3389/fneur.2021.735071 (PMC8733158; doi:10.3389/fneur.2021.735071)

Supplementary Material

# Definition of Healthy Tissue ROIs


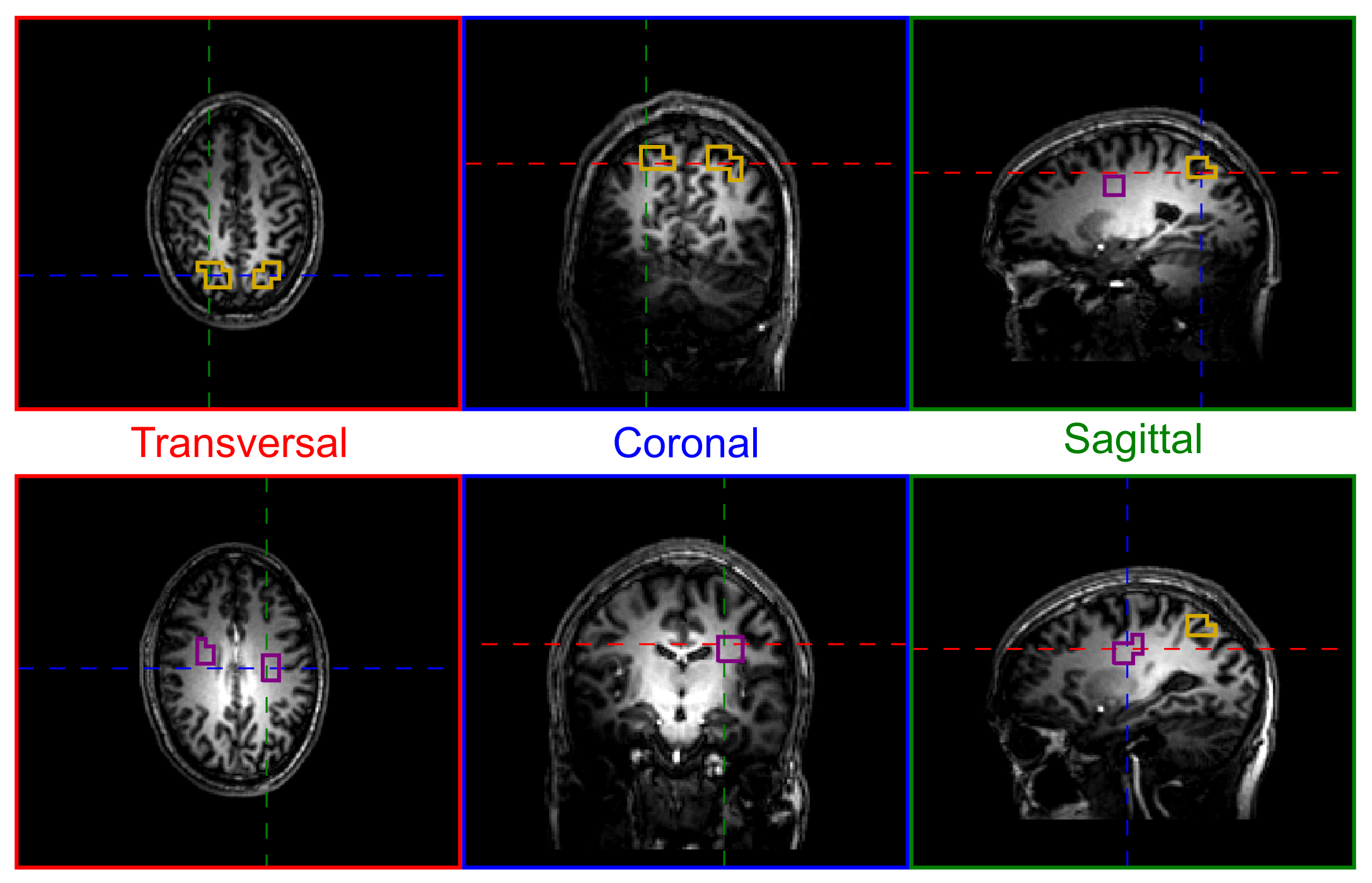


**Supplementary Figure S1.** Representative healthy tissue ROIs in a healthy volunteer, illustrated from two different slice intersection points of 7T T1-weighted MPRAGE images, after mapping onto the ^31^P MRSI grid. The violet and orange volumes indicate the WM and GM ROIs, respectively, which were defined in all subjects at comparable positions. The red, blue, and green lines indicate the positions of transversal, coronal, and sagittal slices, respectively, in each row. Note that the WM and GM ROIs in the patients were only defined on the contralateral side with respect to the tumor.

# Metabolite Maps from the Brain of a Healthy Subject

**
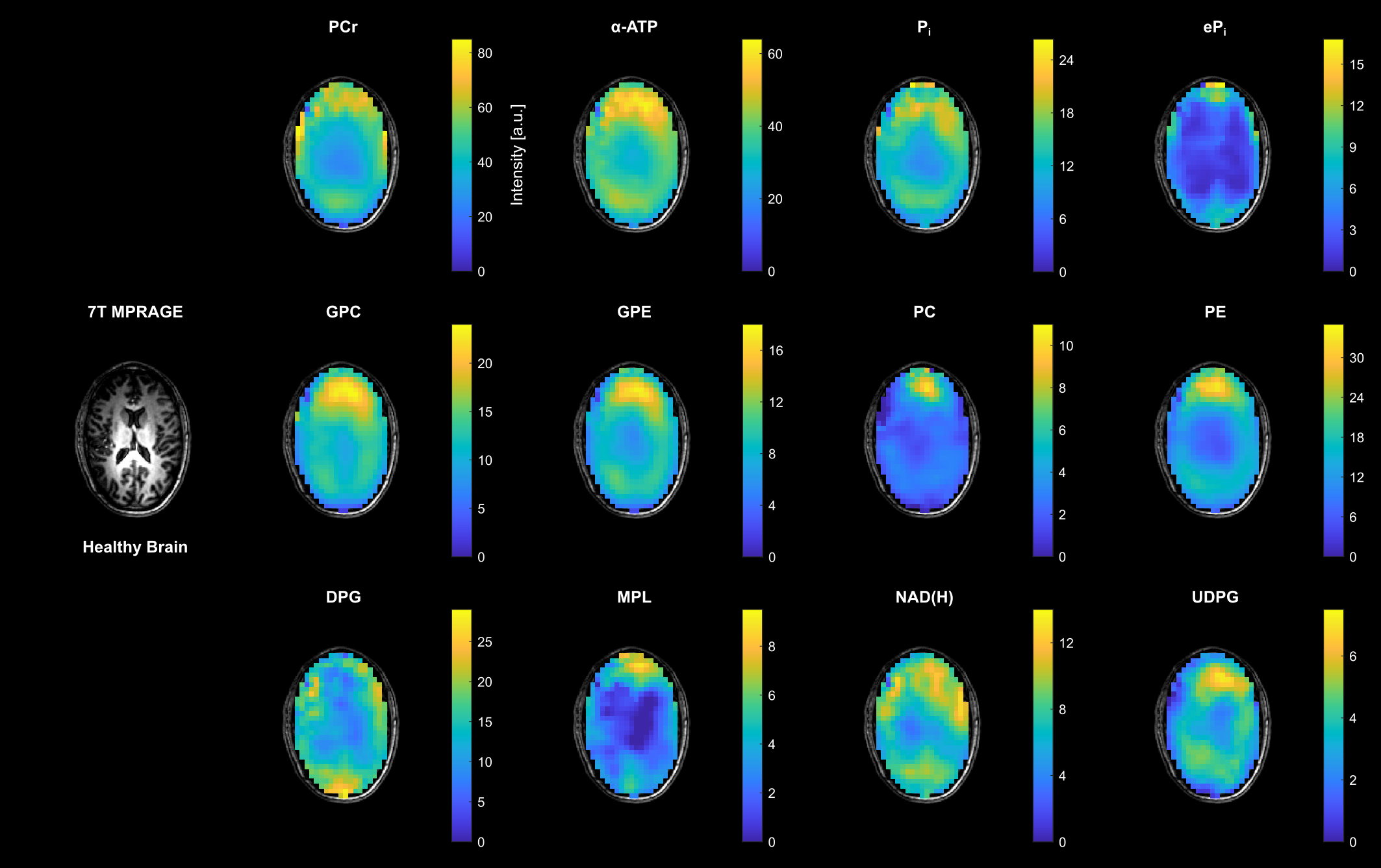
**

**Supplementary Figure S2.** Representative transversal slices of all quantified 3D metabolite maps from a healthy volunteer, shown as intensities in arbitrary units. Note the different scales for the individual metabolites, and that all maps are subject to the coil sensitivity profile. For visual guidance, the metabolite maps are masked to show only brain parenchyma as displayed on the 7T T1-weighted MPRAGE images.

**
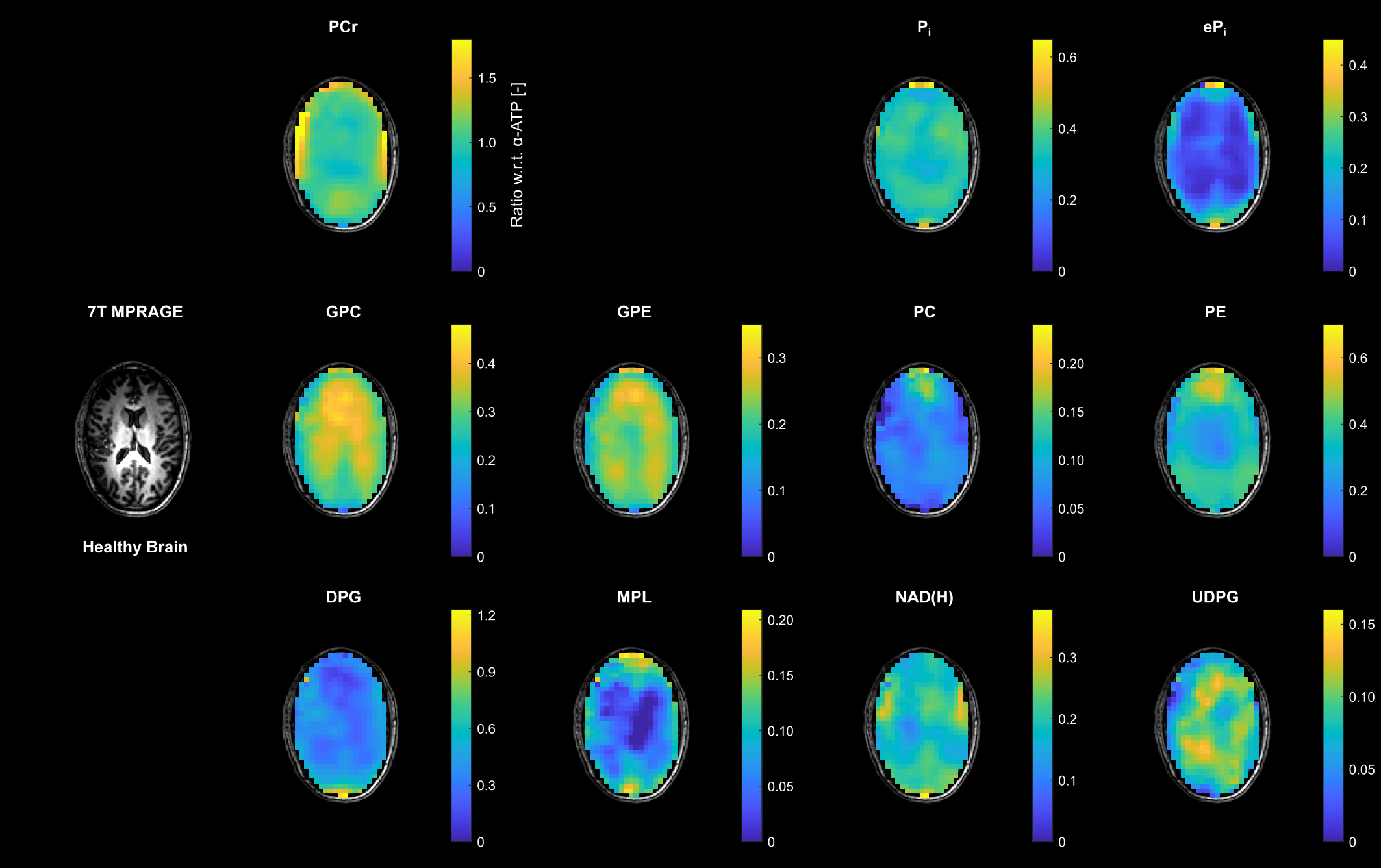
**

**Supplementary Figure S3.** Transversal slices of all quantified 3D metabolite maps (except α-ATP) from the healthy volunteer shown in Fig. S2, given as ratios with respect to the α-ATP intensity. Note the different scales for the individual metabolites. For visual guidance, the metabolite maps are masked to show only brain parenchyma as displayed on the 7T T1-weighted MPRAGE images.

# Metabolite Ratio Maps from Brain Tumor Patients

**
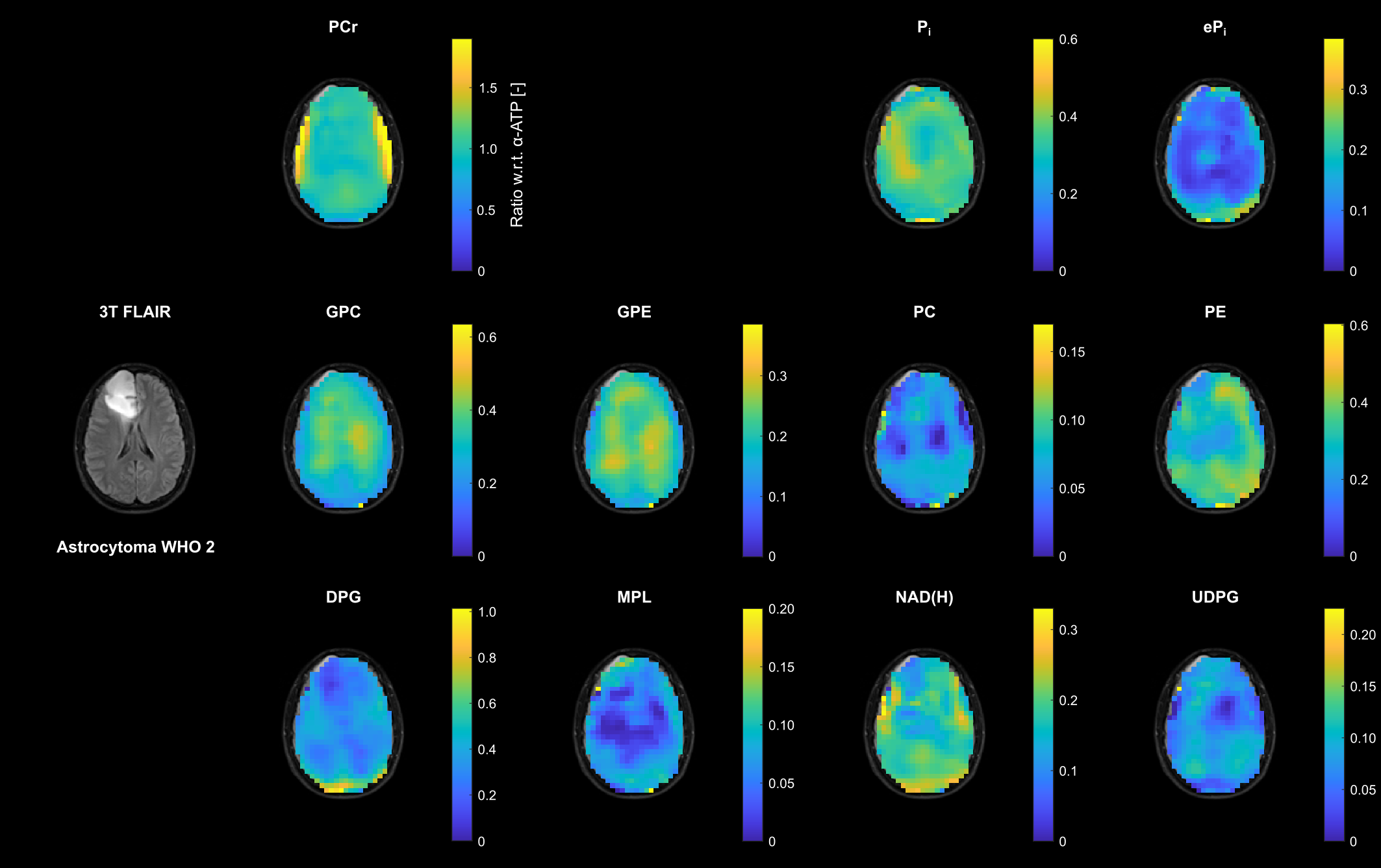
**

**Supplementary Figure S4.** Transversal slices of all quantified 3D metabolite maps (except α-ATP) from the patient with low-grade glioma (astrocytoma WHO 2) shown in Fig. 2, given as ratios with respect to the α-ATP intensity. Note the different scales for the individual metabolites. For visual guidance, the metabolite maps are masked to show only brain parenchyma as displayed on the 3T T2-weighted FLAIR images (coregistered to the MPRAGE images obtained at 7T).

**
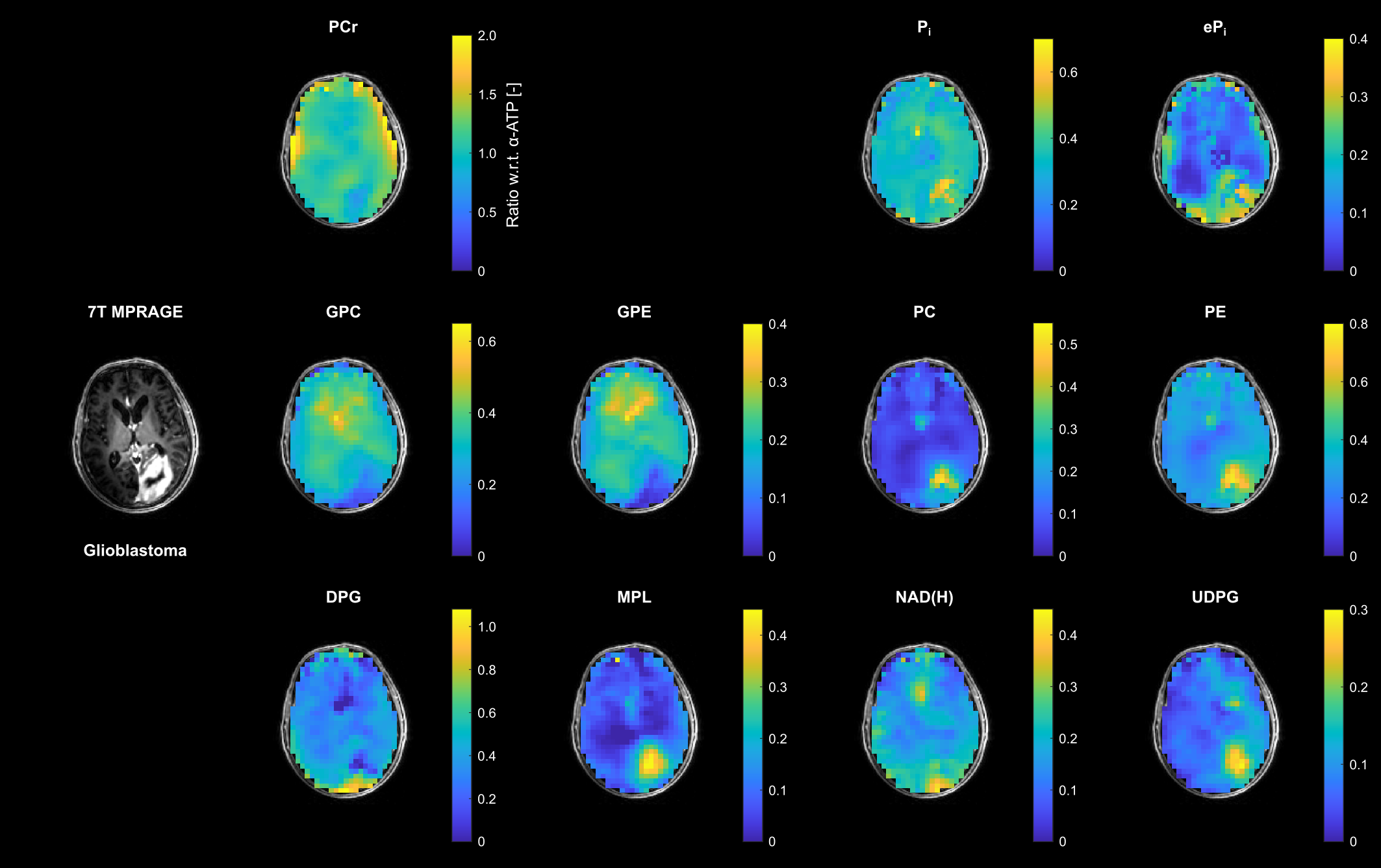
**

**Supplementary Figure S5.** Transversal slices of all quantified 3D metabolite maps (except α-ATP) from the patient with high-grade glioma (glioblastoma) shown in Fig. 3, given as ratios with respect to the α-ATP intensity. Note the different scales for the individual metabolites. For visual guidance, the metabolite maps are masked to show only brain parenchyma as displayed on the 7T non-enhanced T1-weighted MPRAGE images.

# Metabolite-specific ROI Results

**Supplementary Table S6:** **ROI results for PCr of the Study Cohort.** Given are the PCr ratios with respect to the α-ATP intensity as mean ± standard deviation across the segmented ROIs in every subject (cf. Table 2). V1-V3, healthy volunteers; LGG1 & LGG2, astrocytoma, IDH-mutant, WHO grade 2; HGG1, astrocytoma, IDH-mutant, WHO grade 3; HGG2-HGG5, glioblastoma, IDH-wildtype, WHO grade 4.


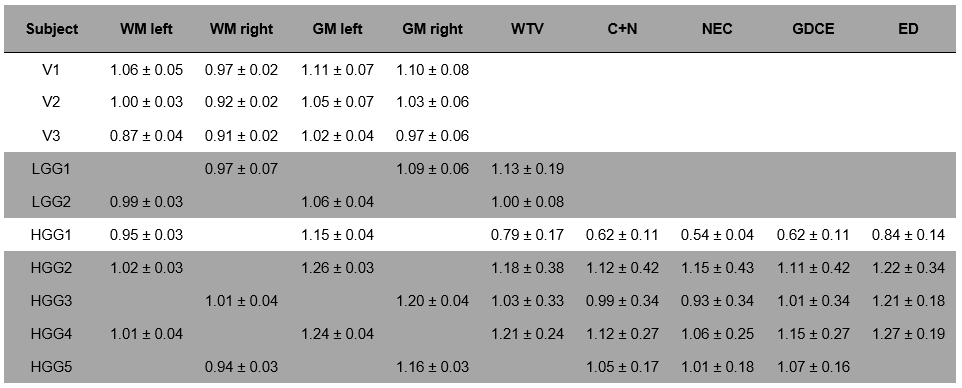


**Supplementary Table S7:** **ROI results for α-ATP of the Study Cohort.** Given are the α-ATP intensities (in arbitrary units) as mean ± standard deviation across the segmented ROIs in every subject (cf. Table 2). V1-V3, healthy volunteers; LGG1 & LGG2, astrocytoma, IDH-mutant, WHO grade 2; HGG1, astrocytoma, IDH-mutant, WHO grade 3; HGG2-HGG5, glioblastoma, IDH-wildtype, WHO grade 4.


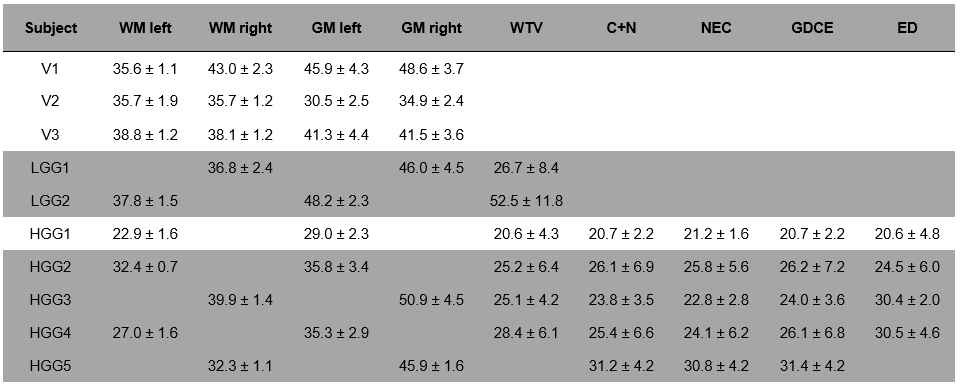


**Supplementary Table S8:** **ROI results for tP_i_ of the Study Cohort.** Given are the tP_i_ ratios with respect to the α-ATP intensity as mean ± standard deviation across the segmented ROIs in every subject (cf. Table 2). V1-V3, healthy volunteers; LGG1 & LGG2, astrocytoma, IDH-mutant, WHO grade 2; HGG1, astrocytoma, IDH-mutant, WHO grade 3; HGG2-HGG5, glioblastoma, IDH-wildtype, WHO grade 4.


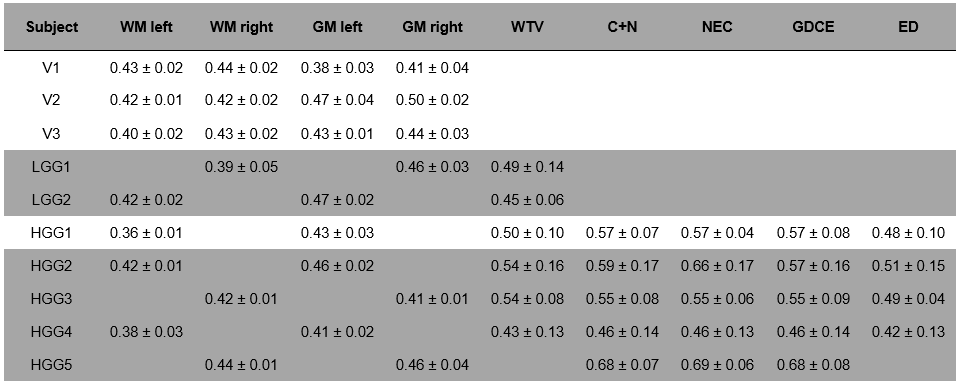


**Supplementary Table S9:** **ROI results for the eP_i_-to-P_i_ ratio of the Study Cohort.** Given are the eP_i_-to-P_i_ ratios as mean ± standard deviation across the segmented ROIs in every subject (cf. Table 2). V1-V3, healthy volunteers; LGG1 & LGG2, astrocytoma, IDH-mutant, WHO grade 2; HGG1, astrocytoma, IDH-mutant, WHO grade 3; HGG2-HGG5, glioblastoma, IDH-wildtype, WHO grade 4.


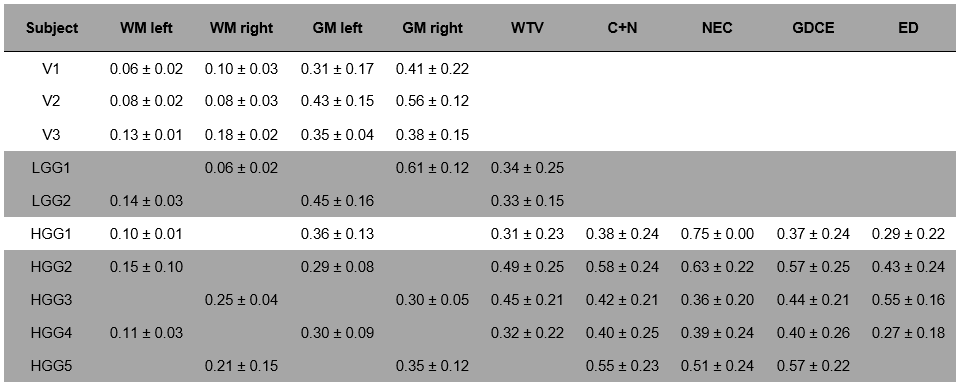


**Supplementary Table S10:** **ROI results for NAD(H) of the Study Cohort.** Given are the NAD(H) ratios with respect to the α-ATP intensity as mean ± standard deviation across the segmented ROIs in every subject (cf. Table 2). V1-V3, healthy volunteers; LGG1 & LGG2, astrocytoma, IDH-mutant, WHO grade 2; HGG1, astrocytoma, IDH-mutant, WHO grade 3; HGG2-HGG5, glioblastoma, IDH-wildtype, WHO grade 4.


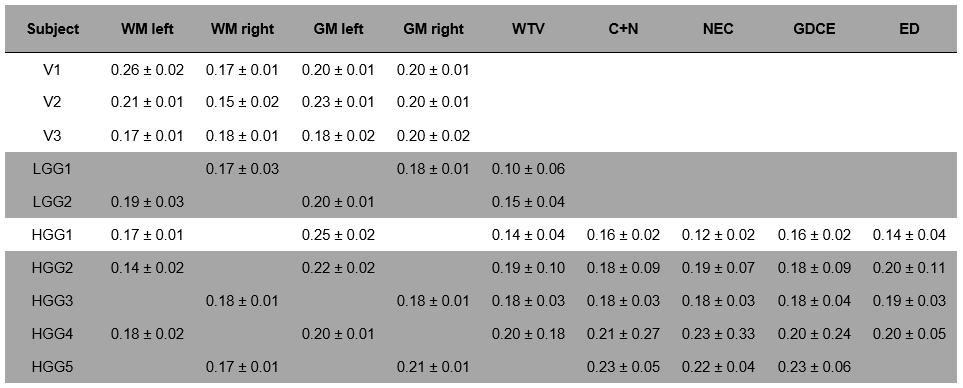


**Supplementary Table S11:** **ROI results for GPC of the Study Cohort.** Given are the GPC ratios with respect to the α-ATP intensity as mean ± standard deviation across the segmented ROIs in every subject (cf. Table 2). V1-V3, healthy volunteers; LGG1 & LGG2, astrocytoma, IDH-mutant, WHO grade 2; HGG1, astrocytoma, IDH-mutant, WHO grade 3; HGG2-HGG5, glioblastoma, IDH-wildtype, WHO grade 4.


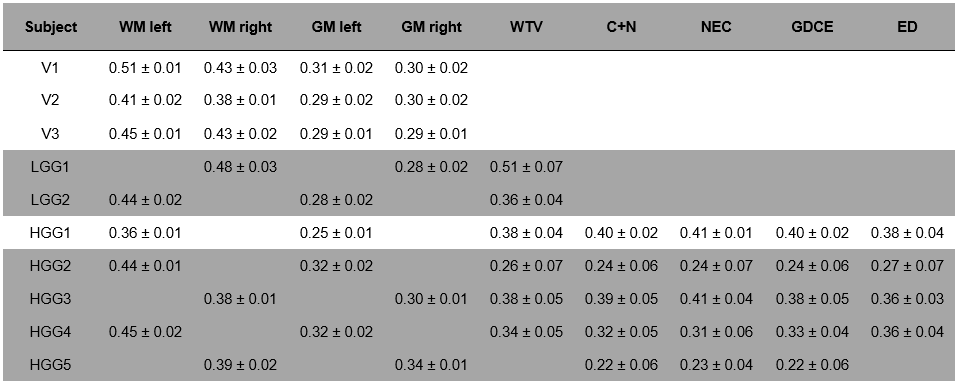


**Supplementary Table S12:** **ROI results for GPE of the Study Cohort.** Given are the GPE ratios with respect to the α-ATP intensity as mean ± standard deviation across the segmented ROIs in every subject (cf. Table 2). V1-V3, healthy volunteers; LGG1 & LGG2, astrocytoma, IDH-mutant, WHO grade 2; HGG1, astrocytoma, IDH-mutant, WHO grade 3; HGG2-HGG5, glioblastoma, IDH-wildtype, WHO grade 4.


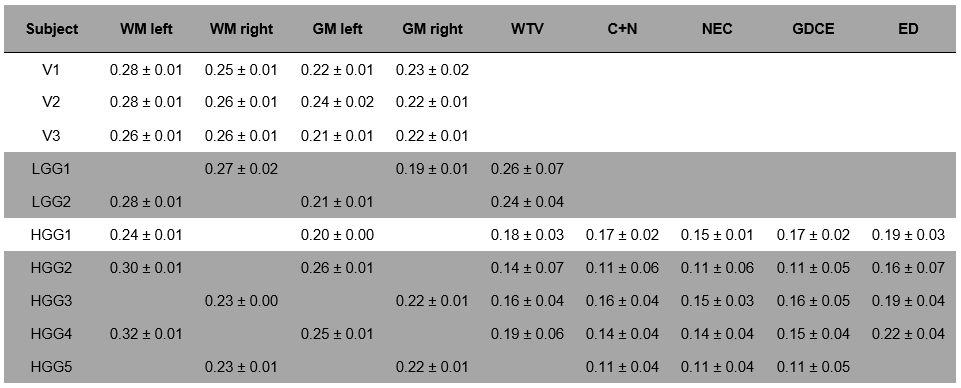


**Supplementary Table S13:** **ROI results for PC of the Study Cohort.** Given are the PC ratios with respect to the α-ATP intensity as mean ± standard deviation across the segmented ROIs in every subject (cf. Table 2). V1-V3, healthy volunteers; LGG1 & LGG2, astrocytoma, IDH-mutant, WHO grade 2; HGG1, astrocytoma, IDH-mutant, WHO grade 3; HGG2-HGG5, glioblastoma, IDH-wildtype, WHO grade 4.


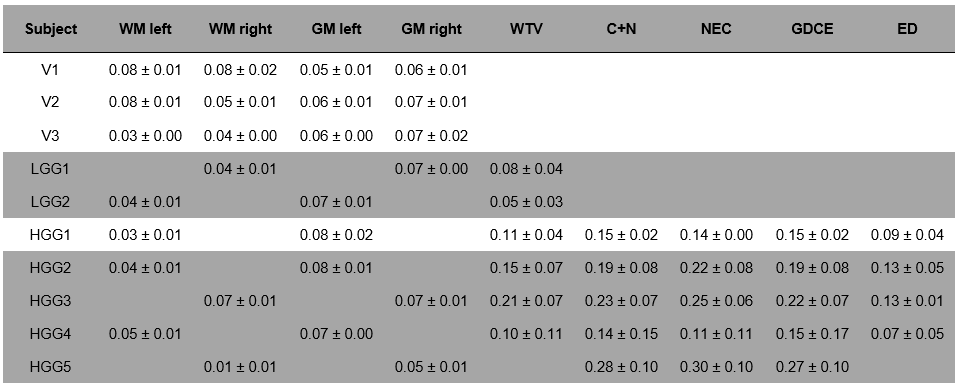


**Supplementary Table S14:** **ROI results for PE of the Study Cohort.** Given are the PE ratios with respect to the α-ATP intensity as mean ± standard deviation across the segmented ROIs in every subject (cf. Table 2). V1-V3, healthy volunteers; LGG1 & LGG2, astrocytoma, IDH-mutant, WHO grade 2; HGG1, astrocytoma, IDH-mutant, WHO grade 3; HGG2-HGG5, glioblastoma, IDH-wildtype, WHO grade 4.


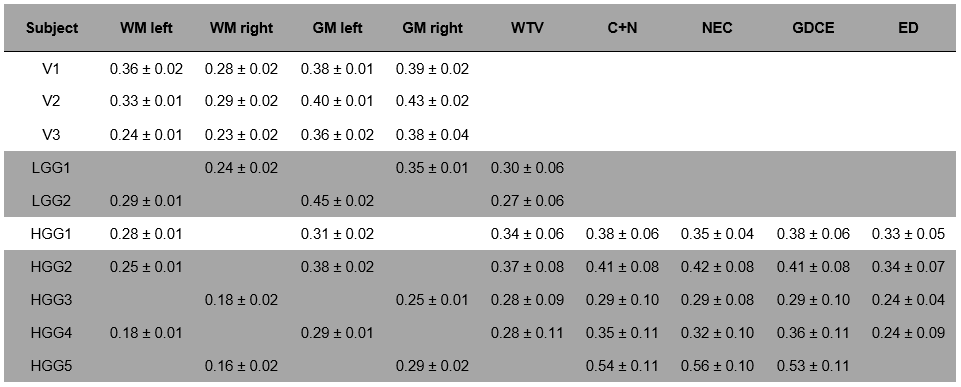


**Supplementary Table S15:** **ROI results for UDPG of the Study Cohort.** Given are the UDPG ratios with respect to the α-ATP intensity as mean ± standard deviation across the segmented ROIs in every subject (cf. Table 2). V1-V3, healthy volunteers; LGG1 & LGG2, astrocytoma, IDH-mutant, WHO grade 2; HGG1, astrocytoma, IDH-mutant, WHO grade 3; HGG2-HGG5, glioblastoma, IDH-wildtype, WHO grade 4.


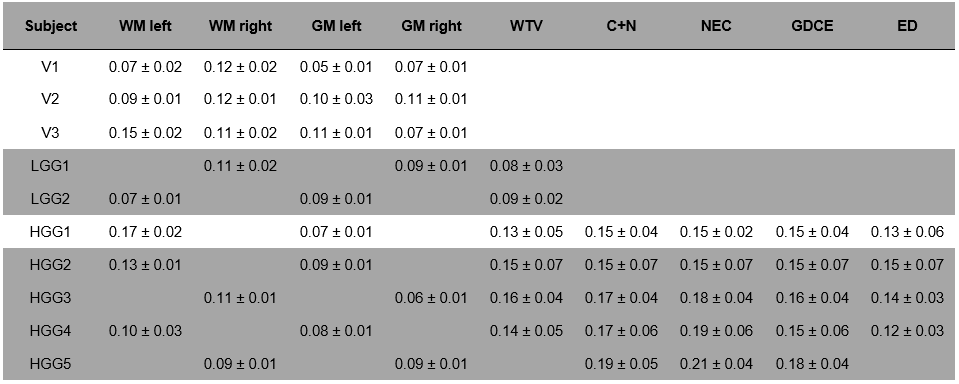


**Supplementary Table S16:** **ROI results for DPG of the Study Cohort.** Given are the DPG ratios with respect to the α-ATP intensity as mean ± standard deviation across the segmented ROIs in every subject (cf. Table 2). V1-V3, healthy volunteers; LGG1 & LGG2, astrocytoma, IDH-mutant, WHO grade 2; HGG1, astrocytoma, IDH-mutant, WHO grade 3; HGG2-HGG5, glioblastoma, IDH-wildtype, WHO grade 4.


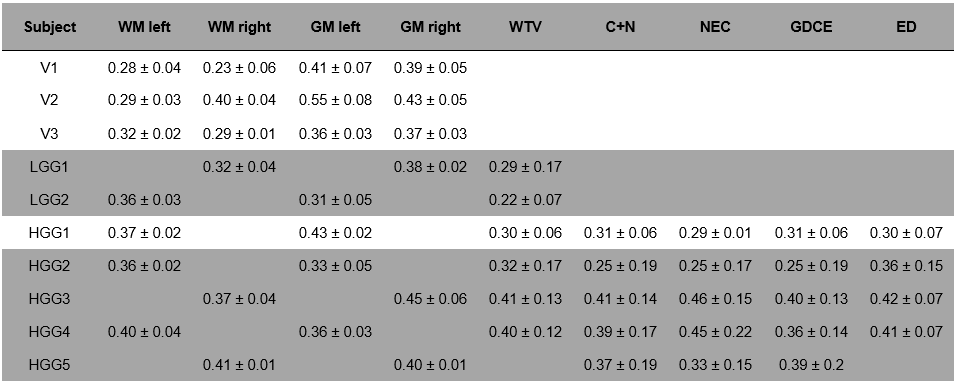


**Supplementary Table S17:** **ROI results for MPL of the Study Cohort.** Given are the MPL ratios with respect to the α-ATP intensity as mean ± standard deviation across the segmented ROIs in every subject (cf. Table 2). V1-V3, healthy volunteers; LGG1 & LGG2, astrocytoma, IDH-mutant, WHO grade 2; HGG1, astrocytoma, IDH-mutant, WHO grade 3; HGG2-HGG5, glioblastoma, IDH-wildtype, WHO grade 4.


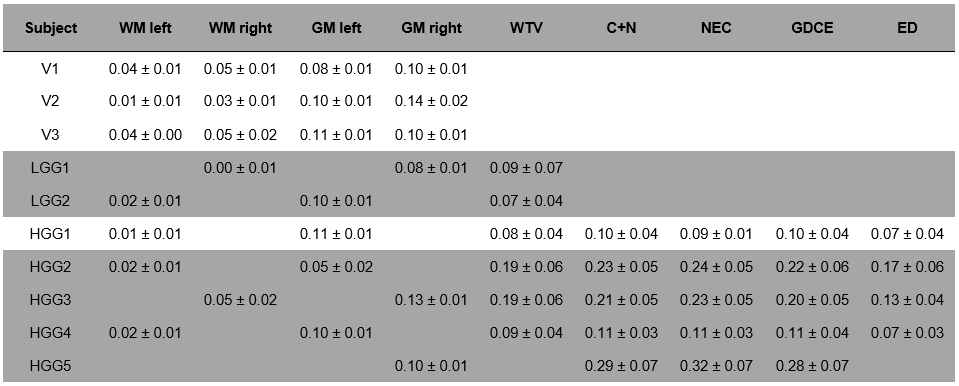

Supplement: Supplementary file 1 [file Data_Sheet_1.DOCX]
